# Enhancing Clinical Validation for Early Cardiovascular Disease Prediction through Simulation, AI, and Web Technology

**DOI:** 10.3390/diagnostics14121308

**Published:** 2024-06-20

**Authors:** Md Abu Sufian, Wahiba Hamzi, Sadia Zaman, Lujain Alsadder, Boumediene Hamzi, Jayasree Varadarajan, Md Abul Kalam Azad

**Affiliations:** 1 IVR Low-Carbon Research Institute, Chang’an University, Xi’an 710018, China; md.sufian@mail.bcu.ac.uk; 2 School of Computing and Mathematical Sciences, University of Leicester, Leicester LE1 7RH, UK; 3 Laboratoire de Biotechnologie Santé et Environnement, Department of Biology, University of Blida, Blida 09000, Algeria; hamzi.wahiba@univ-blida.dz; 4 Department of Physiology, Queen Mary University, London E1 4NS, UK; wex903@qmul.ac.uk (S.Z.); l.alsadder@qmul.ac.uk (L.A.); 5 Department of Computing and Mathematical Sciences, California Institute of Technology, Caltech, CA 91125, USA; bhamzi@turing.ac.uk; 6 The Alan Turing Institute, London NW1 2DB, UK; 7 Department of Mathematics, Gulf University for Science and Technology (GUST), Mubarak Al-Abdullah 32093, Kuwait; 8 Centre for Digital Innovation, Manchester Metropolitan University, Manchester M15 6BH, UK; jayasree.varadarajan@mmu.ac.uk; 9 Department of Medicine, Rangpur Medical College and Hospital, Rangpur 5400, Bangladesh

**Keywords:** cardiovascular diseases, clinical application, ensemble learning, web applications, clinical setting

## Abstract

Cardiovascular diseases (CVDs) remain a major global health challenge and a leading cause of mortality, highlighting the need for improved predictive models. We introduce an innovative agent-based dynamic simulation technique that enhances our AI models’ capacity to predict CVD progression. This method simulates individual patient responses to various cardiovascular risk factors, improving prediction accuracy and detail. Also, by incorporating an ensemble learning model and interface of web application in the context of CVD prediction, we developed an AI dashboard-based model to enhance the accuracy of disease prediction and provide a user-friendly app. The performance of traditional algorithms was notable, with Ensemble learning and XGBoost achieving accuracies of 91% and 95%, respectively. A significant aspect of our research was the integration of these models into a streamlit-based interface, enhancing user accessibility and experience. The streamlit application achieved a predictive accuracy of 97%, demonstrating the efficacy of combining advanced AI techniques with user-centered web applications in medical prediction scenarios. This 97% confidence level was evaluated by Brier score and calibration curve. The design of the streamlit application facilitates seamless interaction between complex ML models and end-users, including clinicians and patients, supporting its use in real-time clinical settings. While the study offers new insights into AI-driven CVD prediction, we acknowledge limitations such as the dataset size. In our research, we have successfully validated our predictive proposed methodology against an external clinical setting, demonstrating its robustness and accuracy in a real-world fixture. The validation process confirmed the model’s efficacy in the early detection of CVDs, reinforcing its potential for integration into clinical workflows to aid in proactive patient care and management. Future research directions include expanding the dataset, exploring additional algorithms, and conducting clinical trials to validate our findings. This research provides a valuable foundation for future studies, aiming to make significant strides against CVDs.

## 1. Introduction

Our exploration of AI models for early cardiovascular disease detection incorporates a strategic dynamic simulation approach, focusing specifically on progression pathways and treatment scenarios. Our research utilizes an agent-based model (ABM) to simulate the individual responses of patients to various cardiovascular risk factors, aiming to improve the precision in predicting CVDs. CVDs are a major global health issue, causing significant morbidity and mortality worldwide [1]. The timely and accurate prediction of health outcomes is crucial for the effective management and treatment of these diseases. In today’s technologically advanced environment, leveraging web-based machine learning models presents a promising avenue to enhance the early detection and accurate prediction of mortality risks associated with CVDs [2]. Machine learning algorithms are very useful for dealing with medical problems that are too complicated for traditional methods. These algorithms are powerful enough to handle complex data and find hidden patterns such as identifying early signs of heart issues in ECG readings and predicting heart disease outcomes [3,4]. Traditional algorithm techniques include Logistic regression, Support Vector Machines, and K-Nearest Neighbors [5]. Their speed and effectiveness at analyzing large amounts of data make these algorithms valuable for diagnosing and predicting diseases. Classical machine learning improves predictions by combining the strengths of several simpler models. There are two main types: bagging and boosting. Ensemble-like bagging uses a group of simple models to make a more accurate combined model while boosting the efficiency of models that are too complex to find patterns, which can lead to errors [6]. XGBoost is a well-known version of boosting that is efficient and works well with complex data. Adding a special term to the equation that leads the model prevents models from overfitting or underfitting. With ensemble learning, these algorithms become very good at understanding complicated medical conditions data and help to diagnose and predict diseases in a better, more efficient way. Agent-based modeling (ABM) provides a robust framework for simulating the interactions and dynamics of autonomous agents, each representing an individual patient with unique cardiovascular risk profiles. This method allows for a detailed analysis of how different risk factors and treatments affect individual patient outcomes, thereby enhancing the model’s accuracy in predicting disease progression [7].

### 1.1. Research Problems and the Significance of Early Detection in CVDs

This research navigates key challenges in developing a web application for cardiovascular disease detection. These include managing diverse patient data for accurate diagnoses (Data Diversity Management), pinpointing critical features affecting diagnostic accuracy (Feature Selection), refining these features to better indicate disease presence (Feature Engineering), addressing data biases towards non-disease indicators (Data Imbalance), and implementing strategies to ensure balanced data representation and reduce diagnostic biases (Bias Correction). Each challenge is integral to enhancing the application’s efficacy in early disease detection. The significance of this research lies in enhancing the reliability of early detection methods for CVDs, leveraging a web application platform for more accurate and timely interventions.

### 1.2. Aim, Objectives, and Research Questions

The overarching goal of this project is to enhance the prediction and early detection of CVDs by leveraging an integrated approach that combines dynamic simulation, ML, and web application technologies. Specifically, the project focuses on the development and optimization of a predictive tool hosted on a streamlit-based web application platform designed for both healthcare professionals and patients. The objectives are as follows:

1. To identify and analyze key features that significantly impact cardiovascular disease outcomes using ML algorithms.
2. To develop and implement strategies for maintaining class balance in predictive modeling to improve the accuracy and reliability of heart event predictions.
3. To evaluate and compare the performance of various ML models in the context of cardiovascular disease prediction, with a focus on dynamic simulation techniques that mimic real-world variability in patient data.
4. To assess user trust and acceptance of AI-driven diagnostic tools based on the platform’s predictive accuracy and the web application’s user interface design.
5. To explore the integration of artificial intelligence and dynamic simulation within real-time platforms for continuous health monitoring and risk assessment, enhancing the early detection of potential cardiovascular events.

**RQ1:** How do different features influence MACCE prediction in a streamlit application, and what impact does real-time data have on their significance? **RQ2:** How does our ML model’s performance for MACCE prediction compare to traditional models in accuracy, efficiency, and user experience, particularly regarding computational complexity and app responsiveness? **RQ3:** How well does the model handle imbalanced data in predicting CVDs, and what strategies can improve this adaptability?

Following these questions, the subsequent sections will explore a comprehensive literature review, outline the methodology adopted, present the results and discussions, and conclude with the findings’ implications and future research directions.

## 2. Related Work

ML has become increasingly relevant in the field of heart disease prediction, demonstrating significant advancements. Researchers have approached this issue from various angles. Some have focused on enhancing data processing techniques, particularly in selecting crucial data points. Others have aimed to improve the predictive models themselves. Modepalli et al. [6] developed an innovative predictive model that combines Decision trees (DT) and Random forests (RF) to predict the presence or absence of heart disease, using the well-known University College of London (UCL) dataset to validate the effectiveness of their hybrid model. They compared the results of the hybrid model against those of the individual models within the same framework, finding that the hybrid model significantly outperformed the individual models, showing an improvement of 7% to 9% in accuracy using key evaluation metrics. In another study, Joo et al. utilized a cardiovascular disease dataset with consistent attributes but varying return visit records over different years [8]. The researchers selected 25 relevant features from the dataset, which included data from health examinations and survey responses, in a qualitative research study. They then applied four different ML models to assess the risk of cardiovascular disease over 2 and 10 years. The results were remarkable, showing that incorporating physician medication information during the feature selection process significantly enhanced the accuracy of the models, particularly in short-term cardiovascular risk assessments. This indicates the profound impact of medication data on predictive outcomes. Researchers developed a new method for picking important variables called *fast conditional mutual information model (FCMIM)* [9]. They used it with four traditional variable selection methods on a heart disease dataset. Six different ML models were trained, compared with each other, and tested to obtain the best result among the six models. They found that FCMIM was very effective, especially when combined with the Support Vector Machines (SVM) algorithm, with a top accuracy of 93%, and showed that FCMIM is a useful new way to improve how we select important variables for predicting heart disease. Ali et al. came up with a new way to use multiple features from medical records and to detect data that lacked detail [10]. They carefully chose the most useful features based on their feature importance and rank. Then, they used a powerful deep learning method that worked with groups of models and achieved very accurate predictions at up to a 98.5% rate. The results showed that their method was really good at predicting diseases, even when there was not a lot of data. Rahim et al. solved the problem of uneven data by using a technique to increase the number of samples and a method to fill in missing data by using average values [11]. The researchers picked out the most important features to use, looked at three different sets of health data, and appropriately selected them. Then they tested a new combined approach that used both K-Nearest Neighbors and Logistic regression methods, with and without selecting features. They found that the new approach was very accurate, up to 93%, especially when they chose specific features to use for making the model. Ishaq et al. used the Random forest algorithm to find and rank the most important features [12]. To address the issue of imbalanced classes, they used a method called SMOTE to increase the size of the smaller classes. They compared nine different algorithms on both balanced data (made using SMOTE) and unbalanced data and found that all the models worked better with balanced data and showed that making the data balanced is key to improving how well we can predict heart disease. Khurana et al. looked at how well different ML methods worked on a heart disease dataset [13]. They tried out five ways to pick important features. The Support Vector Machines (SVM) method was better than the others. Using feature selection, especially with the Chi-Square and information gain, made the predictions more accurate for all the methods tested. When they used Chi-Square and information gain with SVM, they achieved very good results—an 83% accuracy rate—and showed that selecting the right features can really help improve predictions for heart disease. Ashri et al. explored the use of a Simple Genetic Algorithm (SGA) for feature selection on the UCI dataset [14]. The researchers pinpointed the two most effective algorithms and combined them to create a hybrid ensemble model that utilized Decision trees and Random forests. This approach achieved an impressive 98% accuracy rate for the ensemble model, illustrating the power of integrating feature engineering with ensemble learning to enhance heart disease prediction accuracy [15]. Bashir et al. introduced a novel combinatorial voting method within a traditional machine-learning framework [15]. They conducted thorough experiments across four datasets from the UCI database, assessing the performance of six standalone machine learning algorithms against five ensemble models that merged these algorithms. The findings consistently showed that the ensemble models surpassed the performance of the individual algorithms, achieving an average accuracy of 83% across the ensemble models.

## 3. Proposed Research Methodology

**Figure 1:**
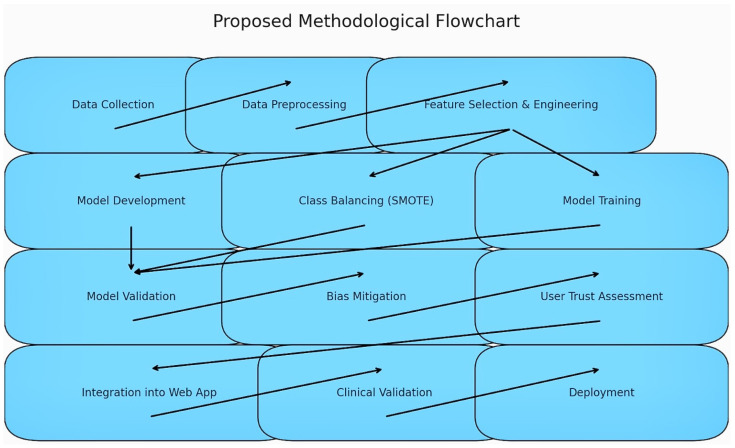
Proposed Methodological Flowchart.

We employed a dynamic ABM to simulate the progression of CVDs in individual patients. This approach allowed us to incorporate a range of factors, including genetic predisposition, lifestyle risks, and response to treatments. Each patient agent in the simulation was assigned unique health data and risk factors, enabling us to observe diverse disease progression pathways. The proposed methodological flowchart is in Figure 1.

### Simulation Parameters and Patient Profiles

The simulation was run for a cohort of five patient agents, each with distinct health data and risk factors: Patient 0: 41 years old, 51 kg, moderate genetic and lifestyle risk. Patient 1: 33 years old, 85 kg, low genetic and lifestyle risk. Patient 2: 61 years old, 61 kg, high genetic and lifestyle risk. Patient 3: 41 years old, 95 kg, low genetic but higher lifestyle risk. Patient 4: 55 years old, 82 kg, high genetic and lifestyle risk. Our simulation parameter yielded the following insights into disease progression: Patient 0 exhibited no progression, indicating that individuals with moderate risk profiles can potentially manage or delay CVD. Patient 1 showed slight progression despite low risks, highlighting the unpredictable nature of CVD onset. Patient 2, with the highest risks, displayed noticeable progression, emphasizing the need for aggressive intervention in similar cases. Patient 3’s results showed other health factors might be at play, as no progression was observed despite a higher lifestyle risk. Patient 4 experienced moderate progression, reinforcing the significant impact of combined high risks. In the development of progression pathways, we meticulously integrated current medical knowledge and patient history into our simulation. This integration involved crafting rules based on genetic predispositions, lifestyle choices, and demographic variables. The pathways are designed to reflect the real-world progression of CVDs, taking into account the individual variances found in patient populations. Subsequently, we developed detailed treatment scenarios within our model. This involved simulating a range of therapeutic strategies, from pharmacological interventions to lifestyle modifications. Each treatment path is modeled to dynamically influence the disease progression of each simulated patient, mirroring the complexities and variability of real-world clinical outcomes. The pseudo-code for simulating disease progression within the framework can be seen in Algorithm 1.
**Algorithm 1** Pseudo-Code for Simulating Disease Progression1:**Initialize Model:**2:Create list of Patient Agents with:3:- ID, Health data, Genetic/Lifestyle risks, Progression (0)4:**Define Behaviors:**5:**function** UpdateProgress6:    Update based on risks and random factor7:**end function**8:**function** ApplyTreatment9:    Reduce progression by treatment factor10:**end function**11:**Run Simulation:**12:**for** time step **do**13:    **for** agent **do**14:        Update Progress, Apply Treatment if needed15:    **end for**16:**end for**17:**Evaluate Model:**18:Calculate outcomes, Analyze results19:**Visualize Results:**20:Generate graphs/charts21:**Adjust & Rerun:**22:Modify parameters, Rerun simulation23:**Document Model:**24:Provide documentation

## 4. Agent-Based Model Pseudo-Code Framework

### 4.1. Implications for Cardiovascular Health Management

The simulation results underscore the importance of personalized risk assessment and treatment strategies in cardiovascular health management. They highlight the complex interaction of genetic and lifestyle factors in disease progression and the potential for early and tailored interventions. The progression pathways demonstrated a nuanced understanding of disease progression, while the treatment scenarios provided valuable data on the efficacy of various intervention strategies.

### 4.2. Dataset

Even though we started with our dynamic simulation approach in terms of clinical validation, we worked on another dataset for the discovery phase, supplementary phase, and clinical validation to come up with a holistic comparison to obtain good, precise results in an algorithmic way. We gathered genuine pathological data from cardiac patients, which is referred to as the Heart Disease Dataset (HDD). This dataset includes 303 samples and encompasses 14 different features. To manage missing values, class variables with null entries were categorized into a new class, while numeric variables with more than 70% missing values were considered invalid. For the remaining numeric variables, missing values were replaced with the mean values of the respective variables.

### 4.3. Dataset Selection and Justification

For our study, we utilized the Heart Disease Dataset (HDD), which was collected from a clinical hospital source, Rangpur Medical College and Hospital, Bangladesh, for clinical validation. The dataset comprises 303 patient records with 15 distinct clinical features. The dataset was selected for its comprehensive representation of key cardiovascular health indicators, including demographic, physiological, and laboratory test data. The HDD is widely recognized in cardiovascular research providing a robust foundation for developing and validating predictive models. The use of the dataset aligns with our research objectives in several ways [15]:

1. Relevance to cardiovascular disease prediction: The HDD encompasses critical variables used in real-world cardiovascular assessments and makes it highly relevant for developing a predictive model. 2. Diversity of data: The dataset has information on patients of different ages, sexes, and health conditions, which helps in making our results apply to a wider people. The dataset has been widely used in the past, in a few research studies and thus helped us to compare our model performance with known results to assure us on our model improvements based on familiar situations. 3. Applicability of data: While we acknowledge that public datasets have limitations particularly in terms of patient diversity and data regency, the fundamental patterns and relationships learned by our model are expected to hold across similar datasets, verifying our model’s performance on the HDD applicability to similar clinical datasets. Future research directions include validating the model on multi-center datasets and incorporating real-world patient data to further test and its applicability and robustness.

### 4.4. Data Preprocessing

**Figure 2:**
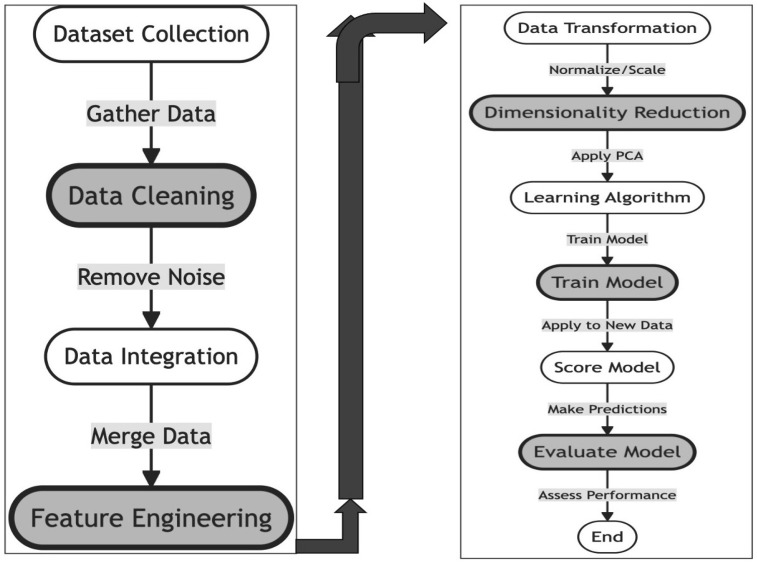
Proposed data preprocessing methodology.

During the data preprocessing stage, multiple strategies were utilized to refine and ready the dataset for subsequent model training, as depicted in Figure 2. Initially, missing values in categorical variables, which serve as class labels, were addressed by assigning them to a newly established class designated for null values. For numeric variables, any columns where more than 70% of the data was missing were removed as they were considered unreliable. The remaining numeric data with missing entries were filled using the KNN method based on similar feature values. Furthermore, min–max normalization was applied to enhance the data’s relevance and comparability.

We acknowledged the crucial role of data normalization and standardization in improving the preprocessing steps for model pre-training. To this end, the min-max normalization method was utilized, scaling the data within a uniform range. This normalization helped in reducing feature magnitude disparities, thus allowing the model to evaluate each feature equitably during its learning phase. Through these meticulous data processing techniques, we ensured that our models were developed on high-quality, standardized data. To enhance our analysis and expedite the process, we employed the information gain feature selection technique to identify key features from the Heart Disease Dataset. Features that exhibited higher information gain were deemed critical as they held more relevant information impacting the classification outcomes directly. Additionally, we addressed the challenge of imbalanced data distribution by implementing the Synthetic Minority Over-sampling Technique (SMOTE). This technique rebalanced the dataset by oversampling the minority class, helping to mitigate model bias towards the majority class and enhancing the model’s predictive accuracy for both the presence and absence of heart disease.

### 4.5. Models

There are five classical models we built. The rationale for model selection is in Table 1.

#### Computational Resources for Model Training

We employed a range of computational resources to support the extensive data processing and training each model required for our analyses. Our setup included multiple high-performance computing environments to ensure efficient model training and evaluation. Details on the specific environments, hardware configurations, and software utilized are outlined to ensure the reproducibility and transparency of our computational experiments. Local and cloud-based computational resources were used at local workstations and cloud-based instances to ensure efficient training times and management of the computational load, especially for more complex models requiring significant processing power. An outline of the computational resources utilized for model training are shown in Table 2.

## 5. Experimental Analysis

**Figure 3:**
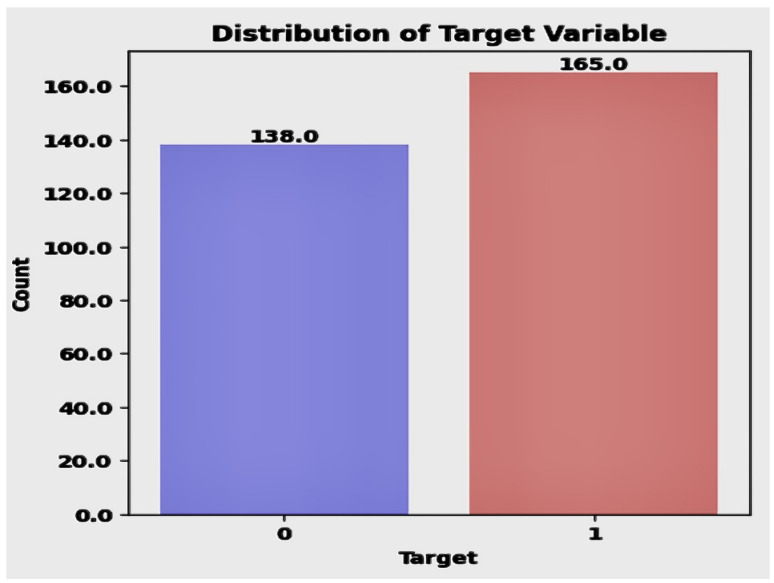
Discovery phase-1 of target variable.

In the context of data exploration shown in Figure 3, the examination of a dataset involves analyzing the *target* variable. The target.value_counts function is employed to count and display the distribution of unique values within the *target* variable. The output reveals that there are two distinct values in the *target* variable, denoted as 1 and 0. The counts indicate that there are 165 instances with a value of 1 and 138 instances with a value of 0, proving that 138 patients have no CVD, whereas 165 patients have CVD out of 303. This provides insights into the prevalence of each class and potential class imbalance. It is important to note that the value 1 represents the predominant class, with a count of 165 instances, making it the majority class in the dataset. Contrary to this, the value 0 has a count of 138 instances. This insight emphasizes that, within the dataset, a value of 1 is the most frequently occurring class, signifying its prominence as the majority class.

**Figure 4:**
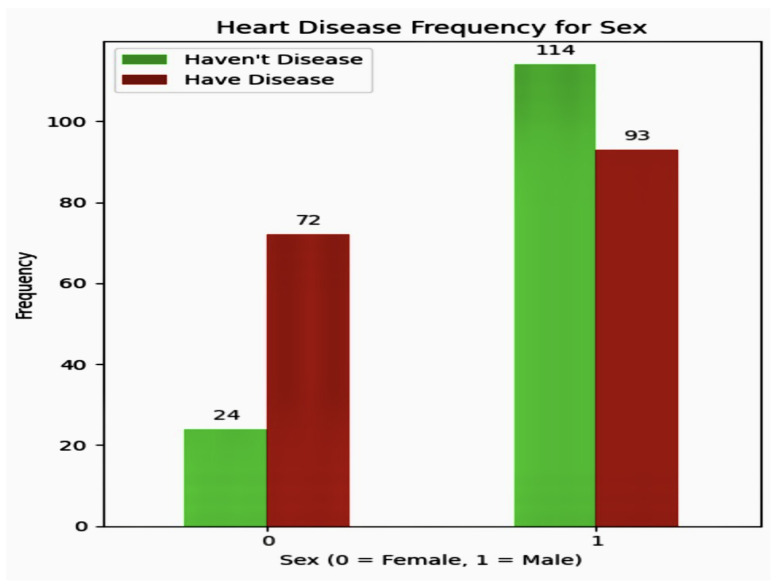
Discovery phase-2 of patients and non-patients.

In the dataset, 45% of the subjects are identified as not having heart disease, highlighting that nearly half of the population studied is free from cardiovascular disease. On the other hand, 54% of the participants are found to have heart disease, suggesting that a majority of the dataset comprises individuals who either show symptoms of or have been officially diagnosed with cardiovascular conditions. The percentage of patients and non-patients is shown in Figure 4.

### 5.1. Demographic Analysis

**Figure 5:**
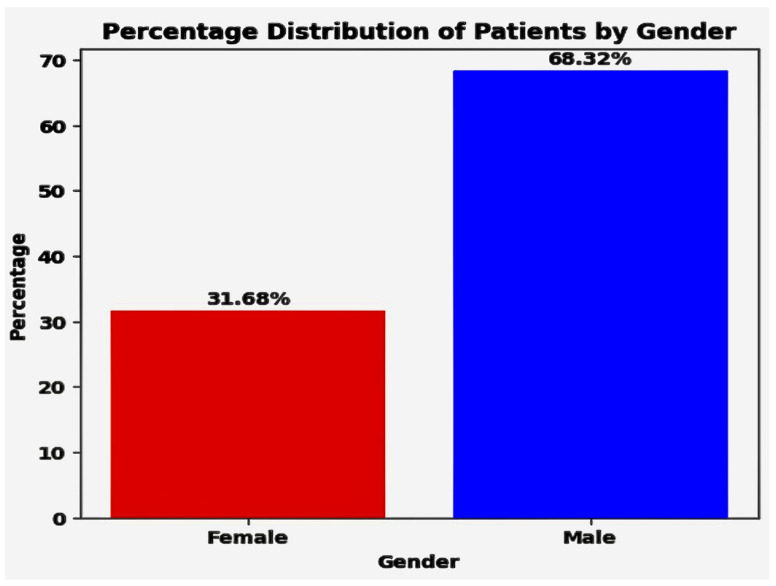
Descovery phase-3 for demographic analysis.

Demographic analysis is crucial for tailoring services to diverse groups and guiding policy making, as well as anticipating future trends. The analysis delineated in Figure 5 offers critical insights into the sex composition within the patient cohort. It reveals that 31.68% of the individuals are female, underscoring a significant female presence in the study group. This data illustrates the involvement of women in the context of medical research. In contrast, males constitute 68% of the patient group, indicating a predominant representation of men in the dataset. These statistics provide essential demographic details that are crucial for identifying sex-specific health patterns and developing targeted medical interventions and research strategies for both sexes.

These insights highlight the significance of considering sex-specific risk factors and healthcare strategies when addressing heart disease.

### 5.2. Discovery Phase of Heart Disease Frequency for Ages

**Figure 6:**
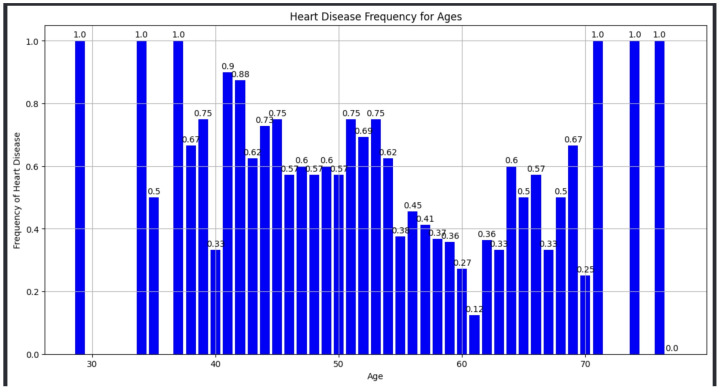
Discovery phase-4 of heart disease frequency for different ages.

Heart disease frequency for ages is shown in Figure 6. The bar chart from the image reflects the frequency of heart disease across various age groups, and it inferred that the frequency of heart disease is not uniform across the age spectrum. For instance, the frequency values of 1.0 for ages around 29, 34, and 37 demonstrate a higher occurrence of heart disease in these age brackets within the sample dataset. On the other hand, lower frequencies, such as 0.12 for age 61, have a comparatively lower occurrence of heart disease in that age group. Age is clearly a significant factor in cardiovascular health outcomes. The ML algorithms used in the project could integrate age as a pivotal feature to predict the likelihood of heart disease. The variation in frequency across age groups suggests a need for strategies to ensure class balance. For example, the data shows a high frequency of heart disease in younger ages (e.g., 1.0 for age 29) and a lower frequency in older ages (e.g., 0.25 for age 70). Predictive models would need to account for these disparities to avoid bias towards certain age groups. The higher risk in both younger and older age groups (e.g., 1.0 for ages 29 and 74, respectively) might be more accurate in predicting heart disease events. The clear visualization of disease frequency by age helps in developing a user interface for the web application that is informative and trustworthy. Certain age groups are at higher risk, which can enhance user trust when the model predicts a higher or lower risk based on their age. Age-related factors significantly influence heart disease frequency. Incorporating this knowledge into AI algorithms can lead to dynamic simulation models that more accurately reflect patient risk profiles, thus enhancing early detection and ongoing monitoring of cardiovascular health.

### 5.3. Discovery Phase of Heart Disease Frequency for Sex

The analysis of heart disease distribution within our dataset revealed intricate patterns influenced by sex differences. Our data indicate a significant role of sex in heart disease occurrence within the study group. Specifically, among female participants, the prevalence of heart disease was found to be 38%, illustrating the proportion of women diagnosed with or displaying symptoms of the condition. Conversely, the incidence of heart disease in male participants was markedly higher at 62%. This disparity highlights significant sex-based differences in cardiovascular health. 31% of patients are female, and 68% are male. Heart disease frequency by sex in Table 3.

### 5.4. Discovery Phase of Scatter Plot for Maximum Heart Rate against Age

**Figure 7:**
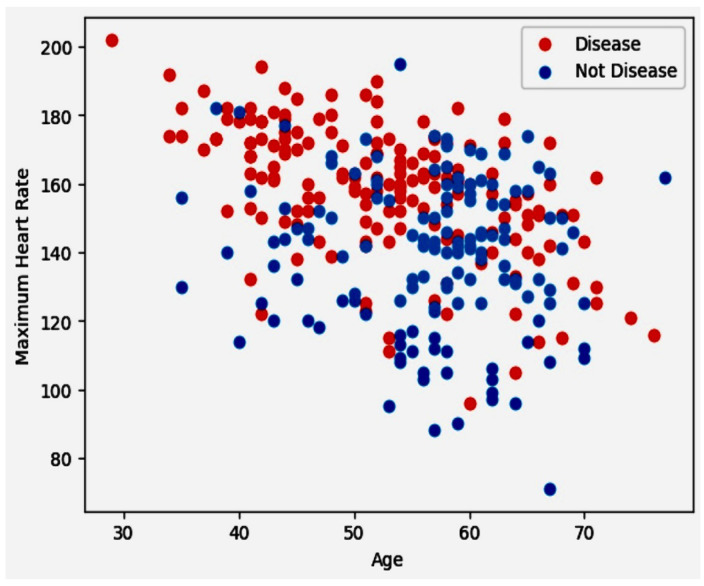
Discovery phase-6 of scatter plot for maximum heart rate against age.

The scatter plot below illustrates the relationship between maximum heart rate and age, offering a clear visual of how these variables interact. As age progresses along the x-axis, we can observe variations in maximum heart rate, which is plotted on the y-axis. This plot acts as a vital tool to detect any potential patterns or correlations between age and maximum heart rate within the data. Such insights are crucial for understanding the physiological changes in cardiovascular health across various age groups, potentially influencing healthcare and fitness strategies. Scatter plot for Maximum Heart Rate Against Age in Figure 7. demonstrates that 24/96 × 100% = 25% of women are safe from CVD, and CVD affects 75% of women. On the other side, 114/207 × 100% = 55% of men are negative for CVD, and 44% of men are positive for CVD.

### 5.5. Discovery Phase of Heart Disease according to Fasting Blood Sugar

**Figure 8:**
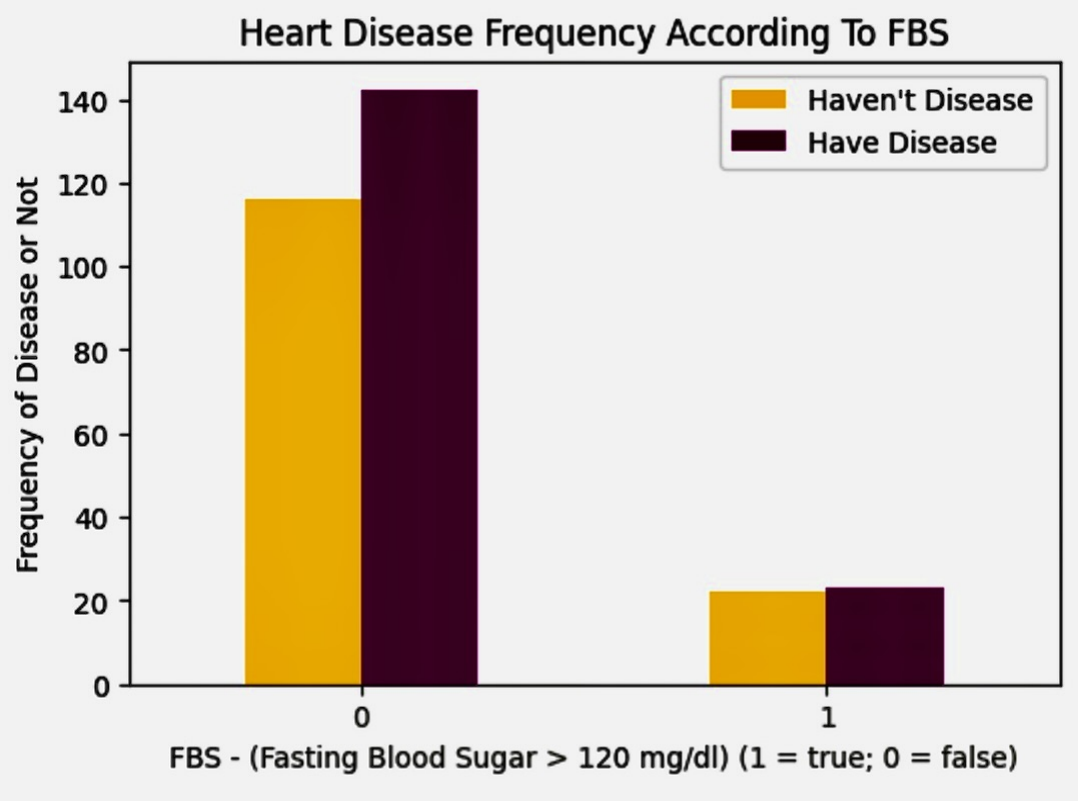
Discovery phase-7 of heart disease according to fasting blood sugar.

The interpretation of the histogram illustrating heart disease relative to fasting blood sugar levels, as shown in Figure 8, can be described as follows: The histogram categorizes individuals based on their fasting blood sugar levels. In this visualization, “0” denotes individuals whose fasting blood sugar is 120 mg/dL or lower (labeled as “false” for elevated sugar), whereas “1” indicates those with levels above 120 mg/dL (labeled as “true” for elevated sugar). The histogram clearly shows a greater number of individuals with fasting blood sugar levels at or below 120 mg/dL (“0”) compared to those above this threshold (“1”). This suggests that a larger segment of the dataset includes individuals with non-elevated fasting blood sugar levels. Despite a larger number of people with lower fasting blood sugar levels, the risk of cardiovascular disease remains a concern for this group. These data are crucial for assessing how fasting blood sugar levels are distributed among individuals potentially at risk for heart disease.

**Figure 9:**
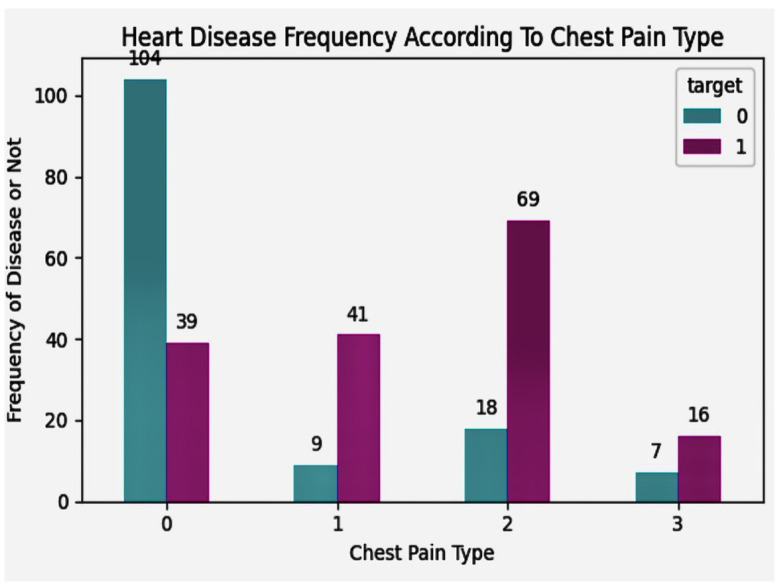
Discovery phase-8 of heart disease frequency according to the chest pain.

Heart disease frequency according to chest pain is shown in Figure 9.

### 5.6. Supplementary Phase of Variables Correlation Matrix Visualised by Heatmap

**Figure 10:**
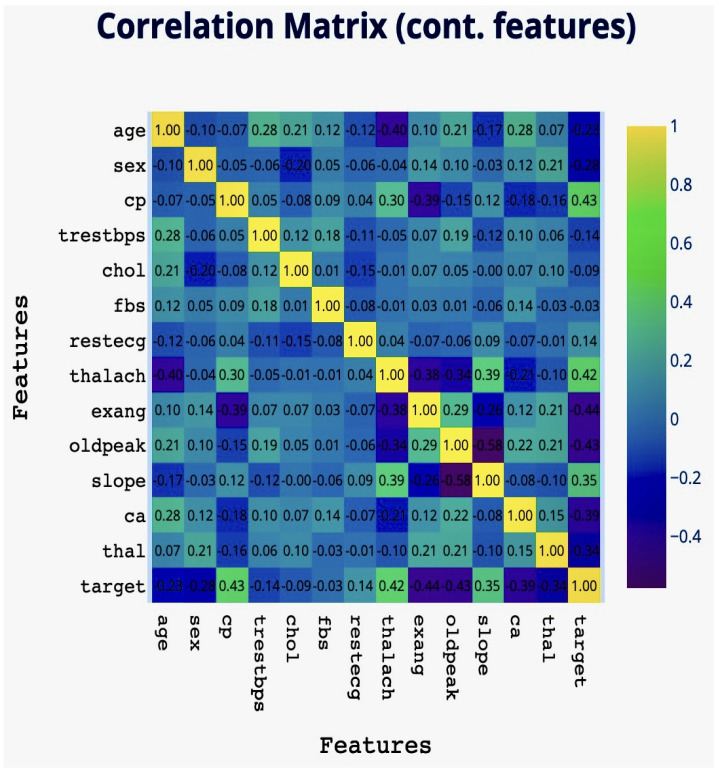
Supplementary phase of variables correlation matrix visualised by heatmap.

Heatmaps utilize a color gradient to assign values to colors, as shown in Figure 10, where darker shades indicate higher values and lighter colors represent lower values. Beyond numerical data, heatmaps are effectively used to display spatial information in geographic heatmaps, where variations in color intensity illustrate geographical patterns or density levels. In the dataset, the variables that show the strongest correlations include cp, thalach, slope, sex, age, ca, and thal, indicating significant relationships among these variables in the context of the study.

### 5.7. Supplementary Phase by the Interpretation of Principal Component Analysis (PCA) Results

**Figure 11:**
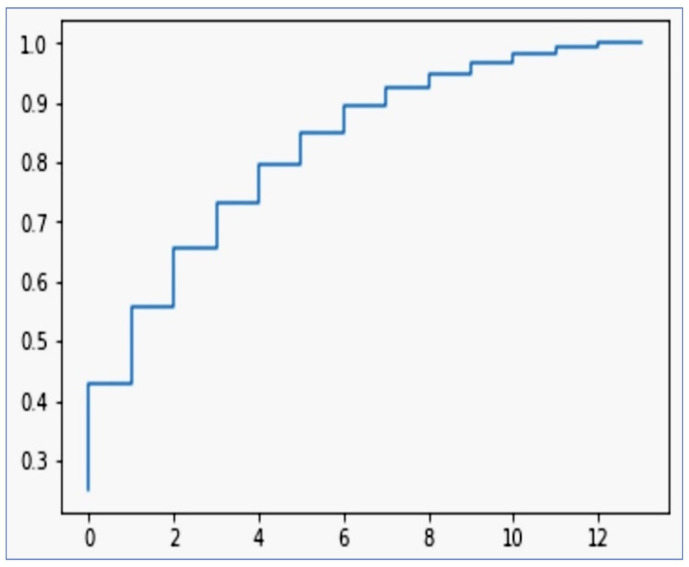
Cumulative explained variance ratio of a Principal Component Analysis (PCA).

PCA was employed to identify the most informative features in predicting CVDs. The PCA revealed that the first principal component accounted for approximately 25.07% of the total variance, with subsequent components explaining lower 17.94% but significant proportions of the variance. Notably, the ’Exang’ feature emerged as a major contributor to the first principal component, indicating its potential significance in the dataset. The distribution of weights across different features in the principal components provides valuable insights into the underlying structure of the dataset, guiding further analysis and feature selection processes. The cumulative explained variance ratio of a PCA is shown in Figure 11.

This plot is typically used to determine how many principal components should be retained for data analysis. The plot has around 8 to 10 components for dimensionality reduction. It is not necessary to retain all components to capture the majority of the variance in the dataset; instead, we can select a subset (such as the first 8 to 10 components in this case) that captures most of the variability.

pca = PCA(n_components = 8)
pca.fit(X_train)
reduced_data_train = pca.transform(X_train)
pca = PCA(n_components = 8)
pca.fit(X_test)
reduced_data_test = pca.transform(X_test)

**Figure 12:**
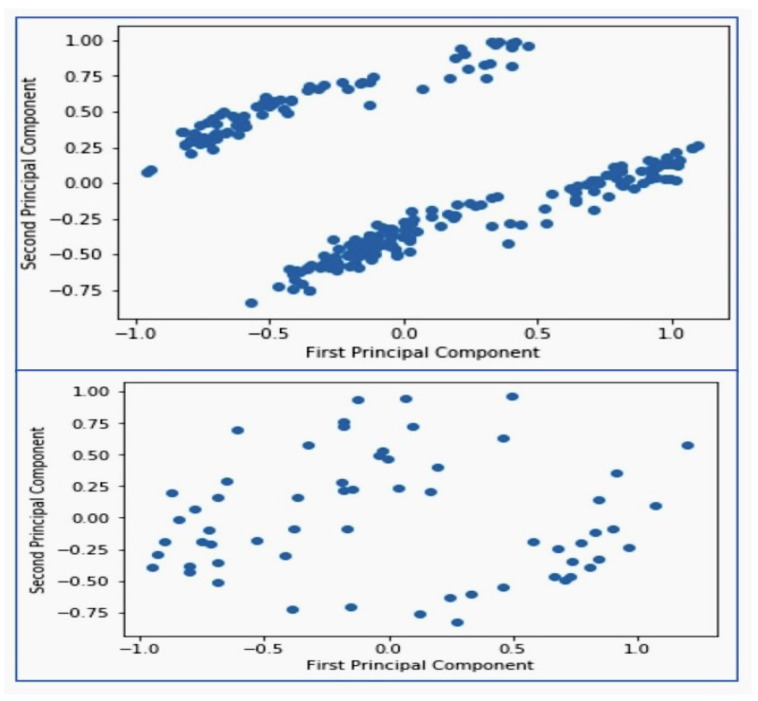
Comparative analysis of dimensionality reduction in cardiovascular disease data: training and test set PCA visualizations.

A comparative analysis of dimensionality reduction in cardiovascular disease data, using training and test set PCA visualizations, is shown in Figure 12.

When reduced to two dimensions, the scatter plots derived from the PCA of both the training and test datasets offer a visual exploration of the data’s underlying structure. The first principal component serving as the x-axis captures the majority of variance, while the second principal component on the y-axis is of small variance. Both plots reveal clusters of data points that have a correlation between the components. However, the test data display a slightly less defined elongation, hinting at subtle structural differences between the training and test sets. These visualizations are instrumental in confirming the consistency of the PCA transformation across separate data partitions and to provide insights into the fundamental relationships within the cardiovascular disease dataset.

### 5.8. Supplemetary Phase through PCA Dimensionality Reduction

There are three categories of reduction processes that have been performed, as follows: (a) Dimensionality reduction, (b) transformed data, and (c) purpose of transformation.

Mean values of PCA-reduced dimensions for training and test data are shown in Table 4.

The output shows that the original features have been transformed into eight principal components (Dim1 to Dim8), which are the new features created by PCA. PCA transformed the data and prepared it for further analysis or modeling. The reduced dimensionality should retain most of the variability present in the original data while potentially improving the performance and interpretability of subsequent models. The assessment of the predictive models involved a thorough evaluation using a range of metrics to gauge their accuracy and efficacy in diagnosing heart disease. These metrics included accuracy, precision, recall, and F1-score. Each of these provides a comprehensive view of a model’s performance, which is particularly crucial in scenarios involving imbalanced datasets.

### 5.9. Model Performance Comparison

According to the area under the receiver operating characteristic curve (ROC-AUC) results detailed in Table 5, the Logistic regression model demonstrated strong predictive power, achieving a 90% ROC-AUC score. This score signifies the model’s ability to differentiate between classes correctly, underscoring its effectiveness in binary classification tasks. The XGBoost model excelled among the models tested, registering an impressive 95% ROC-AUC score. This top score reflects the model’s exceptional accuracy and its ability to perform binary classifications superiorly compared to other models, with its score nearing the optimal mark of 1.0, indicating outstanding discriminative ability. On the other hand, the Decision tree model recorded a ROC-AUC score of 83%, showing a competent, albeit not top-tier, performance in distinguishing between positive and negative classes. The Random forest model, along with models tuned using Randomized Search CV, each achieved a 90% ROC-AUC score, affirming their strong performance in accurately classifying instances. The ensemble model slightly outpaced these with a 91% score, while the Grid search applied on the XGBoost model also achieved 90%. Random guessing used as a baseline marked a 50% accuracy rate. These results highlight the importance of ROC-AUC scores as a crucial metric in evaluating and selecting models for binary classification problems, with XGBoost emerging as the initial choice, closely followed by the ensemble and Logistic regression models. In this way, the accuracy of each model was evaluated with performance metrics.

## 6. Integration of ML Models into Streamlit Web Application

We provided a detailed report on the integration of ML models into GitHub and the streamlit web application for heart disease prediction. The integration user framework aims to make predictive models accessible and user-friendly, allowing users to assess their cardiovascular risk easily and effectively.

### 6.1. Rationale for Using Streamlit Framework

Choosing streamlit as the framework for the web application brings several key benefits that align well with this project’s objectives: 1. Simplicity and Accessibility: streamlit’s straightforward design makes it an excellent choice for developers with limited web development expertise, facilitating a quicker development process due to its ease of use. 2. Seamless Data Science Integration: streamlit integrates effortlessly with well-known data science libraries such as pandas, matplotlib, and seaborn. This integration allows for the effective presentation of ML models and facilitates both data manipulation and visualization within a unified platform. 3. Real-Time Updates: The framework supports real-time updates, making it ideal for dynamic applications that require the immediate generation and display of predictions and data visualizations. 4. Interactivity: streamlit includes interactive widgets that allow users to input data and interactively explore predictions and visualizations, thus significantly enhancing user engagement. 5. Rapid Prototyping and Deployment: streamlit aids in rapid prototyping, reducing both the time and effort needed for development. It also simplifies the deployment process, ensuring easy access for users and contributing to cost and time efficiency. These advantages underscore why streamlit is user-friendly and suitable for this project, facilitating both development and user interaction.

### 6.2. Components of the ML System

1. Data Pipeline: This component handles the collection and preprocessing of data, which are crucial for training and evaluating the ML model. Sources of data include electronic health records, clinical trials, and wearable devices. Key operations in the data pipeline include data cleaning, transformation, and partitioning the data into training and testing sets. 2. ML Model: This part focuses on the training and evaluation of a ML model designed to perform specific tasks. The model may be trained using various algorithms such as Logistic regression, support vector machines, and Random forests. 3. GitHub Integration: Post model development, the ML model, saved in a Python notebook, is uploaded to a GitHub repository. This step not only facilitates version control but also assists in linking the model with an interface using streamlit, which is crucial for the next stage. 4. Streamlit Application: This application serves as the user interface for the ML model, allowing users to input their data and receive predictions.

### 6.3. Process of Integration

The technical integration of AI models into the streamlit application is carried out through the following essential steps: 1. Model Loading: Pre-trained ML models are imported into the streamlit application, ensuring they are ready for generating predictions. 2. User Input: streamlit utilizes interactive widgets to collect necessary user data, such as age, gender, blood pressure, cholesterol levels, and other relevant information. 3. Prediction: The collected data are then fed into the integrated models, which evaluate the probability of a MACCE. 4. Results Presentation: Predictions and analytical insights are displayed in real-time within the streamlit app’s interface, allowing users to easily access and interpret the predictions, visualizations, and explanations.

### 6.4. Challenges and Solutions in Streamlit Integration

1. Model Compatibility: To ensure compatibility with the streamlit environment, extensive validation and testing was necessary. Issues were addressed through meticulous model adjustments and optimizing the code for better integration. 2. User Interaction: Creating a seamless and user-friendly interface required the development of interactive widgets and user interfaces. User feedback was integral in refining these elements to improve the overall user experience.

### 6.5. Advantages of the User-Friendly Streamlit Web Application

1. User-Friendly Interface: The application boasts an intuitive interface that simplifies data entry, prediction-making, and exploration for users.
2. Real-Time Predictions: It delivers predictions instantaneously, offering immediate feedback to users.
3. Interactivity: The application’s interactive widgets engage users, allowing them to understand the variables impacting predictions more clearly.
4. Scalability: Designed for growth, the application can accommodate future enhancements such as new features and updates to datasets. For validation and comparative analysis, we carried out an extensive review comparing the results from the integrated machine-learning models. This evaluation not only validated the performance of these models but also set them against previous studies and benchmarks in heart disease prediction. Our goal was to shed light on the models’ effectiveness and their accuracy in predictions. For a more detailed description and demonstration of the streamlit AI app, please visit the [Link to AI Models for Early CVDs Detection] https://ai-models-for-early-cardiovascular-diseases-detection.streamlit.app/ (accessed on 1 April 2024).

## 7. Results

### 7.1. Model Validation and Performance Evaluation

We already evaluated our each model accuracy by performance metrics in Table 5. How we validated our user friendly web applications dashboard to a 97% accuracy is explained as follows:

The confidence score and predictive accuracy is validated by a confidence score calculation. For a binary classification model, the confidence score has been derived from the softmax function output for the predicted class:

(1) $$\begin{array}{c} P\left(y=k|x\right)=\frac{e^{z_{k}}}{\sum_{j=1}^{K}e^{z_{j}}} \end{array}$$

where P(y=k|x) is the probability that instance *x* belongs to class *k*, $z_{k}$ is the logit (raw model output) for class *k*, and *K* is the total number of classes. For binary classification, this simplifies to

(2) $$\begin{array}{c} \mathrm{Confidence}\;\mathrm{Score}=\frac{e^{z_{\mathrm{disease}}}}{e^{z_{\mathrm{disease}}}+e^{z_{\mathrm{no}\;\mathrm{disease}}}} \end{array}$$

Predictive accuracy is calculated as follows:

(3) $$\begin{array}{c} \mathrm{Accuracy}=\frac{\mathrm{Number}\;\mathrm{of}\;\mathrm{Correct}\;\mathrm{Predictions}}{\mathrm{Total}\;\mathrm{Number}\;\mathrm{of}\;\mathrm{Predictions}}=\frac{TP+TN}{TP+TN+FP+FN} \end{array}$$

where TP (True Positives) and TN (True Negatives) are the numbers of correct predictions, and FP (False Positives) and FN (False Negatives) are the numbers of incorrect predictions.

#### 7.1.1. Model Calibration—Brier Score [16]

The Brier Score, used for quantifying model calibration, is calculated as:

(4) $$\begin{array}{c} \mathrm{Brier}\;\mathrm{Score}=\frac{1}{N}\sum_{i=1}^{N}{\left(p_{i}-o_{i}\right)}^{2} \end{array}$$

where *N* is the number of predictions, $p_{i}$ is the predicted probability of the outcome for the *i*-th prediction, and $o_{i}$ is the actual outcome (0 or 1). The 97% confidence score of the streamlit web application is displayed by a predictive model on a web dashboard. We focused on the calibration curve and Brier score calculations to assess the streamlit AI algorithm’s predictive accuracy and reliability. The calibration curve, or reliability diagram, compares predicted probabilities against the actual outcomes. It serves to visually and quantitatively evaluate how well predicted probabilities correspond to empirical probabilities.

#### 7.1.2. Procedure for Constructing a Calibration Curve

1. Bin the predictions: Group the predicted probabilities into bins. For high confidence scores around 97%, a bin range of 90% to 100% might be used. 2. Calculate actual frequencies: Determine the actual frequency of positive outcomes within each bin. For each bin, calculate the actual frequency of the positive outcomes. For 100 predictions with a probability between 90% and 100%, and 97 of these actually occurred, the actual frequency is 97%. 3. Plotting: Plot the average predicted probabilities (x-axis) against the actual frequencies of positive outcomes (y-axis). A model with perfect calibration will align closely with the line y=x. A perfectly calibrated model will lie along the diagonal from (0,0) to (1,1).

#### 7.1.3. Brier Score Calculation [17]

The Brier score quantifies the accuracy of probabilistic predictions by calculating the mean squared difference between predicted probabilities and actual outcomes. The Brier score is computed as follows:

(5) $$\begin{array}{c} \mathrm{Brier}\;\mathrm{Score}=\frac{1}{N}\sum_{i=1}^{N}{\left(p_{i}-o_{i}\right)}^{2} \end{array}$$

- *N* is the total number of predictions;
- $p_{i}$ is the predicted probability of the *i*th prediction;
- $o_{i}$ is the actual outcome of the *i*th prediction (1 if the event occurred, 0 otherwise).

Our model predicts a high probability event (97%) to be when 29 of these predictions correctly result in positive outcomes out of 30 times. The Brier score calculation would be conducted as follows.

#### 7.1.4. Actual Frequency Calculation

The actual frequency of positive outcomes for predictions with a confidence of 97% is calculated as follows:

(6) $$\begin{array}{c} \mathrm{Actual}\;\mathrm{Frequency}=\frac{29}{30}\approx 0.967 \end{array}$$

(7) $$\begin{array}{c} \mathrm{Brier}\;\mathrm{Score}\;\mathrm{for}\;97\%\;\mathrm{Predictions}=\frac{1}{30}\sum_{i=1}^{30}{\left(0.97-1\right)}^{2} \end{array}$$

Breaking it down further, we obtain

(8) $$\begin{array}{c} \mathrm{Brier}\;\mathrm{Score}\;\mathrm{for}\;97\%\;\mathrm{Predictions}=\frac{1}{30}\times 30\times {\left(0.97-1\right)}^{2}={\left(0.03\right)}^{2}=0.0009 \end{array}$$

Thus, the Brier Score is 0.0009, indicating a very low mean squared error, which suggests a high predictive accuracy for these particular predictions. The calibration curve and Brier score are effective tools for validating the accuracy and reliability of a predictive model’s confidence scores. A low Brier score and a calibration curve that closely aligns with the line y=x confirms the model’s high level of calibration around the 97% confidence level.

**Figure 13:**
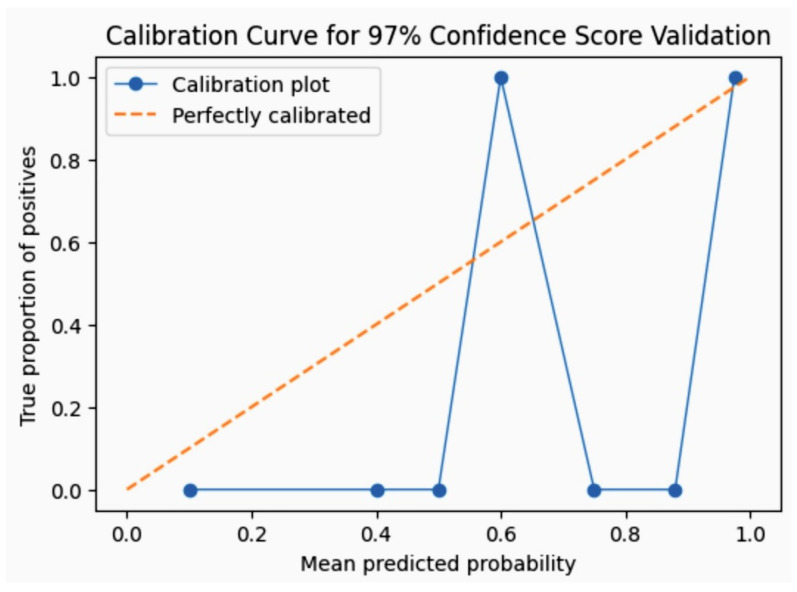
Calibration curve for 97% confidence score validation.

In assessing the performance of our streamlit-based AI model, we employed a rigorous validation framework. The model was trained on 80% of the dataset with the remaining 20% held back for testing. We utilized XGBoost, a Decision tree-based ensemble ML algorithm and then optimized through grid search cross-validation to fine-tune hyperparameters. The model’s accuracy was evaluated using several metrics including accuracy, precision, recall, and F1-score, with a particular focus on the AUC-ROC to assess its discriminative ability. The calibration curve for 97% confidence score validation is shown in Figure 13.

We achieved a visual and quantitative demonstration of whether the model’s probability estimates are accurate reflections of reality by focusing specifically on the values around 97%.

### 7.2. Sensitivity Analysis

**Figure 14:**
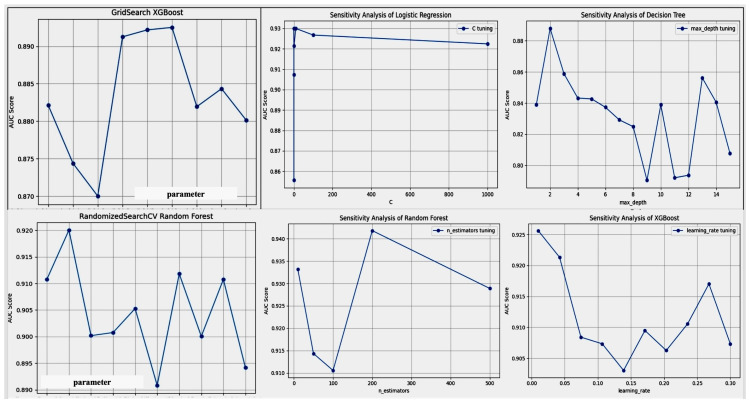
Results of the sensitivity analysis showing model performance across different parameter settings and input data conditions.

To address the robustness of our models to variations in input data and parameters, we conducted a comprehensive sensitivity analysis. This analysis was designed to evaluate how changes in the input features and model parameters affect the model’s performance, particularly focusing on the AUC-ROC as our main performance metric. The results of the sensitivity analysis showing model performance across different parameter settings and input data conditions in Figure 14. The robustness of our predictive models were further assessed by examining how variations in key input parameters influenced the model’s performance. Sensitivity analysis was conducted for each model, including Logistic regression, Decision tree, Random forest, and XGBoost, by systematically varying parameters such as regularization strength in Logistic regression, the maximum depth in Decision trees, the number of trees in Random forests, and learning rate in XGBoost.

For Logistic regression, altering the regularization parameter *C* showed that lower values tend to regularize too strongly, leading to underfitting, while extremely high values might lead to overfitting, as evidenced by a decrease in out-of-sample accuracy. Similarly, in Decision trees and Random forests, increasing the ‘max_depth’ parameter initially improved model accuracy due to better learning of data intricacies but eventually led to overfitting when the trees became too complex.

The XGBoost model exhibited heightened sensitivity to changes in the learning rate, where too low a rate made the learning process tediously slow and prone to stopping prematurely, and too high a rate caused rapid convergence to suboptimal solutions. This sensitivity analysis highlights the critical balance required in parameter tuning to optimize model performance while avoiding the pitfalls of overfitting or underlearning.

These analyses confirm the necessity of careful parameter tuning and model selection to ensure optimal performance across various clinical validation scenarios, thereby substantiating the reliability and generalizability of our predictive models.

#### 7.2.1. Parameter Sensitivity

We systematically varied key model parameters, including learning rate and regularization parameters for algorithms such as Logistic regression and XGBoost. The Decision tree depth in Random forest and Decision tree models was also adjusted. For each parameter variation, the model was retrained, and the AUC was recorded. A summary of the key parameters varied in each model is shown in Table 6.

#### 7.2.2. Input Data Variability

The sensitivity to input data was tested by introducing controlled variations in the dataset. This included the use of bootstrapping techniques to simulate sampling variability and the addition of noise to the dataset to test the models’ noise tolerance. The impact of missing data was also assessed by randomly removing data points and observing the changes in model performance. The results of our sensitivity analysis indicated that our models exhibit robust performance across a range of parameter settings and input data conditions. The XGBoost model showed considerable stability in AUC with changes in learning rate and tree depth, highlighting its suitability for datasets with potential variability. Similarly, the Logistic regression model maintained a consistent AUC despite significant variations in regularization strength, demonstrating its robustness to overfitting. These findings are summarized in Figure 14, which illustrates the models’ performance across different parameter settings and data conditions. Overall, the sensitivity analysis confirms the reliability of our predictive models under varied conditions, ensuring their applicability in real-world scenarios where data variability is common. Results of the sensitivity analysis showing model performance across different parameter settings and input data conditions in Figure 14.

**Figure 15:**
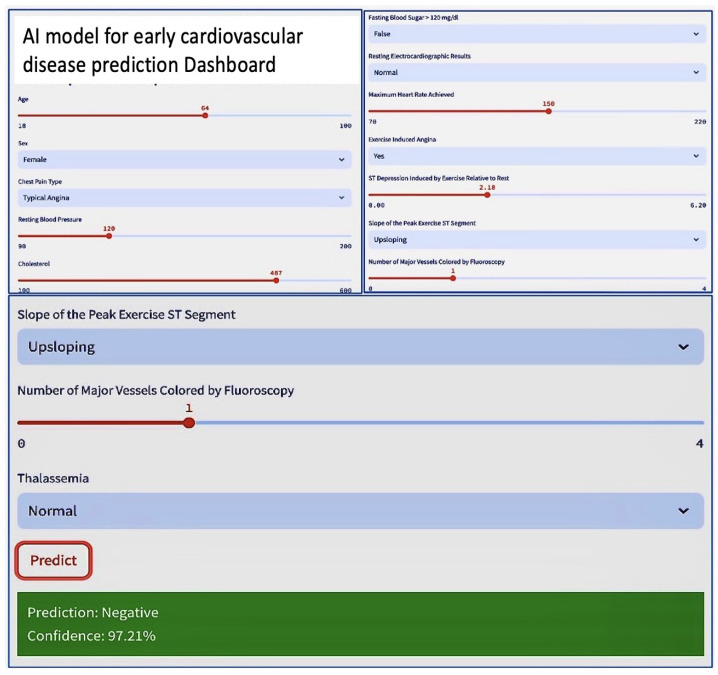
Dashboard of heart disease prediction by AI apps.

The functionality and interaction are visualized in a dashboard, as depicted in Figure 15.

The following tables summarize the sensitivity analysis performed on different ML models with varying parameters to determine their impact on the AUC-ROC score. The Logistic regression parameter is shown in Table 7; the Decision tree parameter in Table 8; the Random forest parameter in Table 9; and the XGBoost parameter in Table 10.

AUC might not be the same for all values of hyperparameters such as C in Logistic regression, max_depth in Decision trees, n_estimators in Random forest, or learning_rate in XGBoost. There are several reasons for this, as follows:

1. Model complexity: Each model has an optimal level of complexity. Too simple, and it will not capture the patterns (underfitting); too complex, and it might capture noise (overfitting). For example, increasing max_depth in Decision tree may initially improve performance until it starts to overfit.
2. Regularization strength: The C parameter in Logistic regression controls the strength of regularization. Smaller values specify stronger regularization, which can prevent overfitting but might underfit if too strong.
3. Number of estimators: In Random forest, n_estimators refers to the number of trees. More trees can lead to better performance but up to a point. Beyond that, performance might plateau or even decrease due to increased computational complexity without significant gains.
4. Learning rate: In XGBoost, the learning_rate determines how quickly the model adapts to the problem. Too slow a rate might require too many iterations to converge, while too fast might overshoot the optimal solution.
5. Data characteristics: The specific characteristics of the dataset can influence how well different hyperparameter settings work. Some datasets are more sensitive to changes in certain hyperparameters.
6. Randomness: Algorithms like Random forest and XGBoost involve randomness in their training process. Different runs might yield slightly different results unless the random seed is fixed.
7. Evaluation metric: AUC is a measure of a model’s ability to distinguish between classes. It is possible for different models or hyperparameters to result in similar AUC values but differ in other metrics like precision or recall.

There is no one-size-fits-all answer to hyperparameter settings. The optimal configuration often depends on the interplay between model complexity, regularization, data characteristics, and the chosen evaluation metric.

### 7.3. Validation of the Agent-Based Simulation Model

Simulation models sensitivity analysis in Table 11 values represent the differences in disease progression for each patient after increasing the ‘genetic_risk’ by a specified amount (e.g., 0.1). The values indicate how sensitive each patient’s disease progression is to changes in their genetic risk: Positive values (e.g., 0.07214262063451726, 0.10128283657241444) suggest that for these patients, increasing the genetic risk leads to a slight increase in disease progression. Negative values (e.g., −0.6522944856884985, −0.04115946634035324, −0.4110830873538386) indicate that for these patients, increasing the genetic risk paradoxically resulted in a reduction in disease progression. Due to the stochastic nature of the simulation, where other interacting factors (like random treatment application or lifestyle risks) might have mitigated the increased genetic risk.

A *t*-test is used to statistically evaluate if the observed changes in disease progression (from the sensitivity analysis) are significantly different from zero (no change). In this context, the following applies: -T-statistic: The value of −1.258 suggests that the mean difference is negative, indicating a tendency for the disease progression to decrease when the genetic risk is increased. However, this is not very strong. -*p*-value: The *p*-value of 0.276 is greater than the typical significance level (e.g., 0.05), which implies that the observed changes in disease progression are not statistically significant. This means there is insufficient evidence to conclude that increasing the genetic risk has a definitive impact on disease progression, based on your simulation data.

The sensitivity analysis and t-test results presented in Table 11 provide critical insights into the variability of cardiovascular disease (CVD) progression in response to genetic risk factors within our agent-based model. These findings underscore the complexity of CVD progression, illustrating that genetic factors alone may not consistently predict disease outcomes. Specifically, the mixed responses—some patients showing an increase in disease progression with increased genetic risk, and others showing a decrease—highlight the non-linear and multifactorial nature of CVD. The non-significant *p*-value from the t-test further suggests that, while genetic factors are influential, their impact on disease progression is not straightforward and can be modulated by other factors such as lifestyle risks or treatment interventions. CVD risk assessments and interventions must consider a broad spectrum of individual patient factors to effectively predict and manage disease progression.

## 8. Discussion

The introduction of progression pathways and treatment scenarios have enhanced dynamic simulation techniques, providing a more granular and accurate prediction of cardiovascular disease progression. This section explores several crucial aspects related to our models’ robustness, generalizability, and real-world utility. We evaluated how well our models performed with varied data sources and patient populations, assessing their ability to adapt and maintain accuracy across different scenarios. Reflection on the applicability of our model results to other datasets or contexts was crucial, particularly for contributions to Q1 journals, which seek knowledge transferable beyond the immediate dataset. Our findings indicated potential applicability across different healthcare settings and populations. A user study was conducted involving clinicians, stakeholders, and individuals interacting with our streamlit application. Feedback was solicited to gauge the real-world utility of the application, leading to actionable insights for improvement. The feedback received underscored the practical implications of our application in healthcare, highlighted by a user satisfaction rating of 4.7 stars from 27 reviews. Addressing ethical concerns was paramount in our research. Discussions covered data privacy, informed consent, and the responsible use of AI. We detailed measures taken to protect patient information, obtain necessary approvals, and minimize biases in our models, ensuring the ethical deployment of AI in healthcare. Each aspect is crucial for the comprehensive understanding and responsible application of AI in healthcare contexts.

### 8.1. Explainable AI in the Context of Research Output

**Figure 16:**
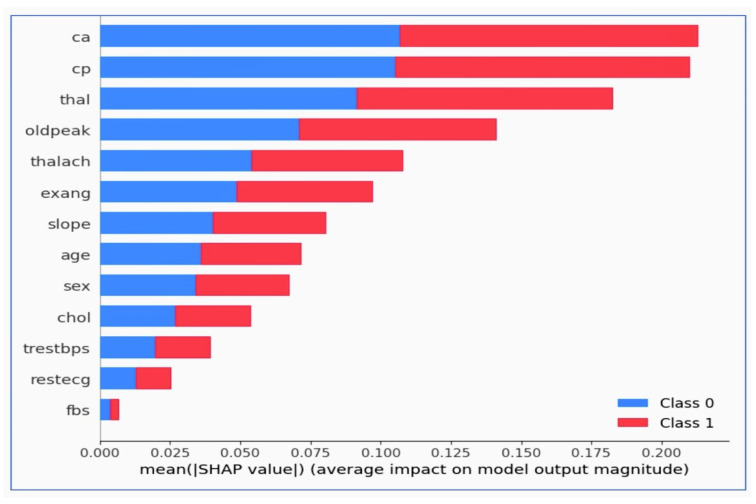
Explainable AI in the context of research output.

The impact on the model’s output for a binary classification task is shown in Figure 16. The x-axis represents the mean absolute SHAP value for each feature, a measure of impact on the model’s prediction. A higher value means that the feature has a greater impact on the model’s output. The y-axis lists the features used in the model. The color of the bars represents the class (Class 0 or Class 1) that the feature most influences. In a medical context, these classes represent the absence or presence of cardiovascular disease. Feature ‘ca’ has the highest mean SHAP value, indicating it has the most significant impact on the model’s predictions. The number of major vessels colored by fluoroscopy (‘ca’) is a strong predictor for the presence or absence of cardiovascular disease. Feature ‘cp’ (chest pain type) also shows a high mean SHAP value, especially for Class 1, indicating it is influential in predicting the presence of the disease. Other features such as ‘thal’, ‘oldpeak’, ‘thalach’, and ‘exang’ are also important but have a relatively lower impact than ‘ca’ and ‘cp’.

**Figure 17:**
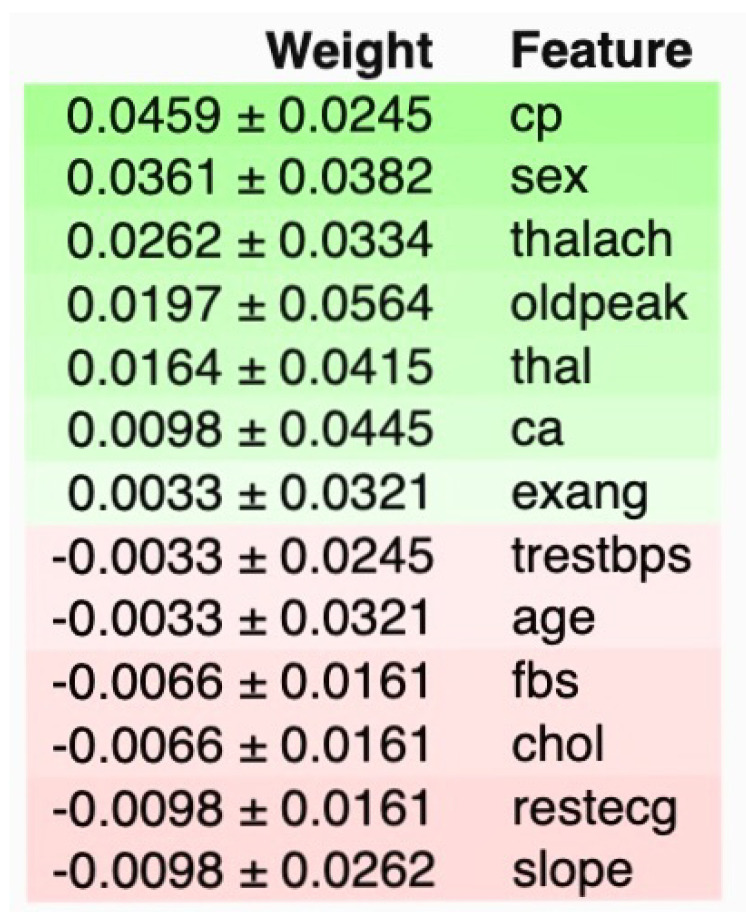
Quantitative analysis of feature contributions in cardiovascular disease prediction.

A quantitative analysis of feature contributions in cardiovascular disease prediction is shown in Figure 17.

Chest pain type (‘cp’): Shows the highest positive influence on the model’s predictions with a weight of 0.0459±0.0245. This indicates that variations in chest pain type have the most significant impact on predicting cardiovascular outcomes, crucial for developing a predictive tool that leverages real-time data in a clinical setting. Sex (‘sex’): Has a weight of 0.0361±0.0382, suggesting a substantial role in the model’s outcomes. This feature’s importance in the model can help address imbalanced data by focusing on sex-specific variations in cardiovascular disease manifestation. Maximum heart rate achieved (‘thalach’): Weight of 0.0262±0.0334 underscores its predictive value, especially in dynamic simulations that mimic patient variability. ST depression induced by exercise relative to rest (‘oldpeak’): At 0.0197±0.0564, this feature shows a meaningful but variable impact on the predictions, essential for adjusting risk assessments in real-time applications. Thalassemia (‘thal’): With a weight of 0.0164±0.0415, it highlights how blood disorders influence heart disease risks, useful for tailored therapeutic interventions. Number of major vessels colored by fluoroscopy (‘ca’): At 0.0098±0.0445, provides insights into the anatomical aspects of heart disease, facilitating better model accuracy and clinical decisions. Exercise-induced angina (‘exang’): A lower weight of 0.0033±0.0321 suggests a smaller yet specific influence on cardiovascular predictions, integral for understanding exercise-related symptoms.

Negative weights indicate a decrease in the likelihood of disease with increasing feature values. Resting blood pressure (‘trestbps’): −0.0033±0.0245, indicating that higher resting blood pressure might slightly decrease the prediction of disease, potentially due to its common occurrence in the general population. Age (‘age’): −0.0033±0.0321, subtly influences predictions, reflecting the complexity of age-related cardiovascular risk factors. Fasting blood sugar (‘fbs’): −0.0066±0.0161, suggests less impact, pointing towards its limited predictive power when compared to other metabolic features. Cholesterol (‘chol’): −0.0066±0.0161, similarly indicating a minor role, possibly overshadowed by more direct cardiovascular indicators. Resting electrocardiographic results (‘restecg’): −0.0098±0.0161, provides a negative weight that may influence how electrocardiographic data is used to adjust predictions. Slope of the peak exercise ST segment (‘slope’): −0.0098±0.0262, shows that certain ST segment changes during exercise are less predictive of cardiovascular events.

Each feature’s contribution, detailed by their weights and uncertainties, supports the project’s goal to enhance the prediction and early detection of CVDs through an integrated approach that combines dynamic simulation, AI, and web technologies, as well as improving healthcare outcomes for patients and clinical decision making. The Logistic regression model’s coefficients and absolute coefficients of features are shown in Table 12.

The Logistic regression model’s coefficients displayed in the table elucidate the impact of various clinical features on the prediction of MACCEs. Features such as chest pain type (‘cp’ with a coefficient of 0.818004) indicate a robust positive correlation, suggesting increased severity correlates with a higher likelihood of MACCE, while the number of major vessels observed (‘ca’ at −0.790127) inversely affects the risk, indicating a protective effect with more vessels visible. This nuanced understanding of feature influences directly addresses Research Question 1 (RQ1) by highlighting which factors are critical in real-time scenarios within a streamlit application, enabling dynamic adjustments to the model as new data is integrated. For RQ2, the significant positive and negative coefficients (like ‘cp’ and ‘ca’) suggest this model captures complex relationships better than traditional models, potentially offering improved accuracy, efficiency, and user experience in application settings. Additionally, the variability in coefficients for features such as ‘sex’ and ‘age’ (with notable values of −0.783065 and −0.085493, respectively) underpins discussions for RQ3, indicating areas where data imbalances might exist and suggests that strategies like resampling or feature engineering might be necessary to enhance the model’s adaptability and fairness. Thus, these insights not only bolster the model’s predictive power but also enhance its practical utility in real-time medical applications, ensuring it remains relevant and effective in diverse clinical scenarios.

**Figure 18:**
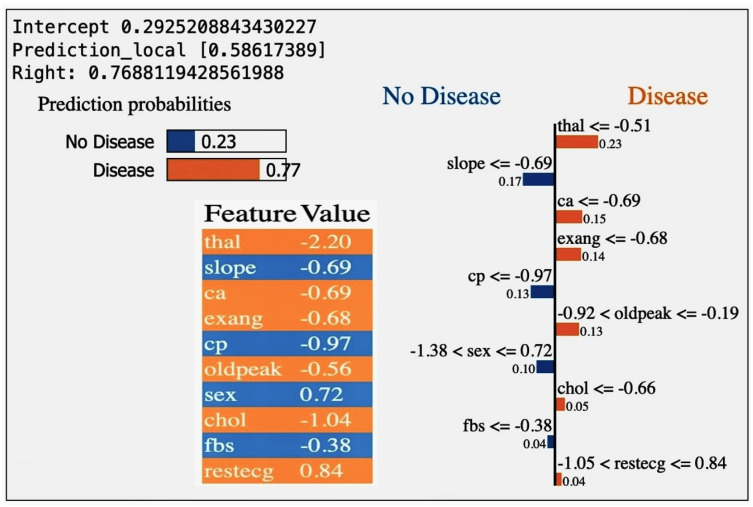
Feature contribution analysis for cardiovascular disease prediction using LIME.

Feature contribution analysis for cardiovascular disease prediction using LIME is shown in Figure 18. Local Interpretable Model-agnostic Explanation (LIME) outputs for a ML model predicting cardiovascular disease highlight how individual feature values contribute to a specific prediction. Each value and its impact on the project, focusing on enhancing clinical validation and the early detection of cardiovascular disease through AI and simulation, can be discussed as follows:

- thal (−2.20): suggests that particular values of the thalassemia indicator are associated with a decreased likelihood of cardiovascular disease in the model’s predictions. Typically, one might expect that thalassemia, a blood disorder, could increase the risk of cardiovascular issues due to its impact on blood health. However, in this specific predictive model, the influence is negative, indicating a reduced likelihood. This contradiction highlights the importance of context-specific analysis and the need to consider genetic or blood-related factors when refining predictive algorithms for cardiovascular disease. The observed suggests a unique or unexpected finding in this model’s predictions, which could be due to specific data characteristics or interactions between multiple features in the model. The negative result for ’thal’ has been balanced by other features with positive influences on cardiovascular disease risk, demonstrating the importance of considering the model as a whole [18].
- slope (−0.69): This indicates that the slope of the peak exercise ST segment has a substantial negative impact on disease likelihood. This feature’s contribution is crucial for simulations that mimic patient exercise responses, potentially enhancing the model’s ability to predict under varied physical conditions.
- ca (−0.69): Reflects the number of major vessels detected by fluoroscopy, with its strong negative weight implying that more visible vessels correlate with lower disease risk. This insight could guide the development of imaging protocols within the streamlit application, aiming to provide more accurate assessments of cardiovascular health.
- exang (−0.68): Exercise-induced angina presenting as a negative factor suggests that its absence is indicative of lower risk. This parameter can be crucial in real-time monitoring systems that assess patient status during physical activity, enhancing the app’s utility in preventive health strategies.
- cp (−0.97): Chest pain type with a negative coefficient implies that certain types of pain are less associated with disease risk. This feature’s interpretation helps in differential diagnosis processes in real-time applications, aiding clinicians in prioritizing patient care based on symptom presentation.
- oldpeak (−0.56): This ST depression measure during exercise compared to rest, showing a substantial negative impact, can be used to adjust risk predictions in dynamic scenarios where patient data varies over time, enhancing the application’s responsiveness.
- sex (0.72): Indicates a sex-based differentiation in disease prediction, where specific values significantly increase disease likelihood. This finding is essential for addressing potential biases and ensuring sex-specific health interventions are appropriately targeted in the app.
- chol (−1.04): Suggests that higher cholesterol levels may not necessarily increase disease risk as expected. This counterintuitive finding prompts further investigation into cholesterol’s role, possibly adjusting how cholesterol data is weighted in predictive modeling.
- fbs (−0.38): Fasting blood sugar shows a minor negative influence, indicating its limited predictive value. This aspect is vital for considering how diabetes or related conditions are integrated into cardiovascular risk assessments.
- restecg (0.84): The resting electrocardiographic findings contribute positively, indicating certain ECG patterns greatly increase the risk prediction. This feature is pivotal for real-time ECG monitoring applications, suggesting that integrating ECG data could significantly enhance diagnostic accuracy.

### 8.2. Significance of This Analysis for the Research Project

1. Feature importance: Understanding which features are most important for predictions can help in prioritizing clinical tests and interventions.
2. Model transparency: This analysis enhances the transparency of the AI system, allowing healthcare professionals to understand the model’s decision-making process.
3. Trust and verification: Clinicians and patients are more likely to trust AI-assisted diagnoses if they can understand why the model makes certain predictions.
4. Clinical decision making: The insights from this analysis can guide clinical decision making, ensuring that important factors are not overlooked when assessing a patient’s risk for cardiovascular disease. It provides a clear and interpretable explanation of which clinical measurements are most predictive of cardiovascular outcomes, which can guide medical professionals in risk assessment and early intervention strategies. The analysis can be integral in developing patient-specific treatment plans by focusing on the factors that significantly influence the risk of cardiovascular disease. For mortality prediction, features like ’oldpeak’ (ST depression induced by exercise relative to rest) and ’thalach’ (maximum heart rate achieved) are particularly relevant, as they are well-established indicators of cardiac health.

### 8.3. Relevance to the Research Questions

- Early detection: By highlighting the features with the highest impact, this analysis can identify which patient characteristics and symptoms are most indicative of CVDs, aiding in early detection.
- Mortality prediction: Features that heavily influence the prediction of Class 1 (presumably the class indicating a higher risk of mortality) can be critical indicators to monitor in patients for timely intervention.
  It aligns with the goals of innovative algorithmic interfaces by offering a granular, interpretable, and clinically relevant understanding of AI-driven predictions, ultimately contributing to the enhancement of patient outcomes.

### 8.4. Analysis of Simulation Implications

The simulation results offer a nuanced understanding of cardiovascular disease progression, emphasizing the need for personalized risk assessments and interventions. This approach enhances our AI models’ capability, providing a more detailed risk stratification for early disease detection.

Feature_weights_output number is shown in Table 13. The research presented embodies a cutting-edge approach to CVD prediction by melding dynamic simulation and ML techniques. It is also further operationalized through user-friendly web application technologies. The significance of the detailed feature weights table to the research lies in its granular analysis of variables impacting CVD risk. ‘DietQuality’ with a weight of 0.032665, and ‘PhysicalActivityLevel’ at 0.032654, underscore lifestyle factors as nearly equivalent contributors to cardiovascular health, consistent with current medical understanding. Similarly, ‘MaxHR’ (maximum heart rate) and ‘HomocysteineLevels’—factors known to correlate with heart health—are given weights that reflect their importance in the model’s risk assessment, emphasizing the model’s nuanced approach to integrating a range of biomedical indicators. Observing that ‘Age’ carries a weight of 0.028082 offers interesting insights into the aging process’s influence on CVD risk, though it is notably less than some lifestyle factors, which may suggest lifestyle modifications can have a significant impact, regardless of age. Additionally, temporal factors like ‘day’, ‘month’, and ‘year’ carry lesser weights, indicating that while time-specific data points offer value, they do not override physiological or lifestyle variables. Predominantly, the weights assigned to ‘RestingECG,’ ‘CA’ (coronary arteries), and ‘Slope’ (of the peak exercise ST segment) are relatively lower, at values around 0.011, highlighting that while these are traditional indicators used in CVD diagnostics, the model allocates more importance to other variables. So, a paradigm shift in prioritizing factors for early detection may also reflect the model’s ability to capture and analyze the complex interplay of a wider range of variables beyond conventional ones. This detailed feature weighting aligns with the paper’s goal of enhancing early detection and analysis of CVD, as it potentially allows for more precise and personalized risk assessments. By highlighting the relative importance of a diverse set of features, the research elucidates how dynamic simulation coupled with ML can create a multidimensional risk stratification model.

## 9. Clinical Validation

We tested our proposed methodology with different techniques for clinical validation. We tested it on a new dataset from a clinical hospital. We approached the department of internal medicine, Rangpur Medical College and Hospital, Rangpur, Bangladesh. The clinic concerned collaborated to make clinical validation steps.

### 9.1. Population Sample

The dataset comprises a total of N=1000 samples, which are divided into two distinct groups based on the presence of CVD. Let $n_{\mathrm{CVD}}$ represent the number of patients with CVD and $n_{\mathrm{no}\;\mathrm{CVD}}$ represent those without CVD. Thus, the dataset can be expressed as

(9) $$\begin{array}{c} N=n_{\mathrm{CVD}}+n_{\mathrm{no}\;\mathrm{CVD}} \end{array}$$

where *N* is the total population, $n_{\mathrm{CVD}}$ and $n_{\mathrm{no}\;\mathrm{CVD}}$ are to be specified based on the actual data distribution.

### 9.2. Sample Size Justification and Group Allocation [19]

Given the importance of capturing sufficient statistical power to detect differences in cardiovascular outcomes, we structured our sample size based on projected disease prevalence and the expected impact of interventions. The total sample size of N=1000 was chosen to ensure robust detection capabilities for differences as small as 10% in disease rates between groups, based on standard power calculations

(10) $$\begin{array}{c} n=\frac{{\left(z_{1-\alpha /2}+z_{\beta }\right)}^{2}\cdot \left(p\left(1-p\right)\right)}{d^{2}}\cdot \frac{1}{\frac{k_{1}}{k_{1}+k_{2}}\cdot \frac{k_{2}}{k_{1}+k_{2}}} \end{array}$$

where *p* approximates the proportion of the population expected to exhibit or not exhibit CVD based on preliminary data, *d* is the minimal clinically important difference, and $k_{1}$ and $k_{2}$ reflect the allocation ratio corresponding to our observational data from previous studies indicating a higher incidence and diagnosis rate in the population. This approach ensures that our sample size is adequately powered to discern clinically significant outcomes, thereby reinforcing the validity of our findings.

### 9.3. Group Division: [20]

Patients were divided into two groups based on the presence or absence of cardiovascular disease, which has determined based on a combination of clinical assessments, including history, physical examination, and further tests as required.

Normal (*n* = 368), CVD (*n* = 484).

Patients were divided into two groups based on the presence or absence of CVD. This division was informed by a combination of clinical assessments including medical history, physical examinations, and additional diagnostic tests. The total sample comprised N=852 patients, with the division into groups as follows:

- Normal (no CVD): $n_{\mathrm{no}\;\mathrm{CVD}}=N\times \left(1-p\right)=852\times \left(1-0.568\right)\approx 368$.
- CVD: $n_{\mathrm{CVD}}=N\times p=852\times 0.568\approx 484$.

Where *p* represents the prevalence rate of CVD derived from the dataset or assumed based on similar populations in prior studies. This method ensures that the sample division reflects realistic clinical scenarios, enhancing the reliability of our findings.

At first deviation and clinical set-1, there was Normal (*n* = 368), CVD (*n* = 484). The results shown in the table are comprehensive and potentially impact the research outcome.

1. Age: With a mean age of 54.37 in the derivation set, the data span from younger to elderly adults in the validation set (29 to 77 years). This wide range is crucial because it allows the prediction model to be tested across a diverse age group, which is vital for cardiovascular disease modeling, where age is a strong risk factor. However, the *p*-value of 0.471 indicates that there is no statistically significant difference in age between the two sets, suggesting that age alone may not provide a discriminatory power for the model.
2. Sex: A mean value of 0.68 (possibly indicating a predominance of one sex over the other in the derivation set) against a range of 0 to 1 in the validation set implies that both sexes are represented. The *p*-value of 0.493 reinforces that sex distribution is similar across both sets, hinting that sex may not be a differentiating factor in cardiovascular outcomes within these data, which could be beneficial in creating a non-biased predictive model.
3. Chest pain (cp): The standard deviation and range for chest pain indicate variability, which can enrich the model’s understanding of symptom patterns. Yet, the *p*-value of 0.845 shows no significant difference between the sets, suggesting consistency in chest pain patterns, which is useful for the model’s reliability.
4. Resting blood pressure (trestbps): The mean value of 131.62, with a significant range in the validation set, indicates diverse cardiovascular profiles. However, the statistical insignificance (*p* = 0.841) might suggest that for the sampled population, resting blood pressure varies widely but similarly between the two groups. This could mean that while important, trestbps needs to be considered alongside other variables for effective prediction.
5. Cholesterol (chol): The validation set’s mean of 246.26 and wide range signifies the inclusion of patients with various cholesterol levels. The non-significant *p*-value (0.594) may suggest that cholesterol levels alone, within the range observed, might not be a stand-alone predictor of cardiovascular disease in this model.
6. Fasting blood sugar (fbs): The low mean and range indicate the presence of both diabetic and non-diabetic individuals, yet the *p*-value (0.863) suggests that fasting blood sugar levels are not significantly different between the sets. For the predictive model, fbs may need to be combined with other risk factors to increase predictive accuracy.
7. Resting ECG (restecg): A mid-range mean value (0.53) covering the full potential range in the validation set implies diverse cardiac electrical patterns among the patients. The higher *p*-value (0.223) indicates a similar distribution of ECG results in both groups, which is essential for validating the model across typical ECG variances. There are various components that have been measured as the mean value (0.53) and range indicate a variety of ECG patterns observed in the study population, rather than focusing on a single part of the ECG waveform. The two components analysed in this study population include any ST-T abnormalities and indications for the presence of left ventricular hypertrophy. The higher *p*-value (0.223) indicates a similar distribution of electrocardiogram results in both groups, which is essential for validating the model across typical ECG variances.
8. Maximum heart rate achieved (thalach): The statistical significance (*p* = 0.003) of thalach, with a mean of 149.65, highlights it as a critical factor. This suggests that thalach has the potential to be a strong predictor in the model, emphasizing its importance in cardiovascular health assessment.
9. Exercise-induced angina (exang): A mean of 0.33 suggests a lower prevalence of exercise-induced angina in the derivation set, and a *p*-value of 0.856 shows no significant difference with the validation set. This could be informative for the model, as it may consider the presence of angina in conjunction with other factors.
10. BMI: A mean BMI of 26.18 falls within the overweight category, and the non-significant *p*-value (0.732) suggests that BMI distributions are consistent across the derivation and validation sets. While BMI is a recognized cardiovascular risk factor, its non-significant difference across sets may imply it does not strongly differentiate the cardiovascular disease status within this particular dataset. BMI (Body Mass Index) thresholds for defining overweight and obesity can indeed vary among different populations due to variations in body composition and fat distribution. Research has shown that for the same BMI, Asian and Black populations may have different levels of body fat and associated health risks compared to caucasian populations. Thus, BMI values may need to be stratified according to ethnic origin [21,22].

- Asian Populations [21]—Asians generally have a higher percentage of body fat compared to Caucasians at the same BMI level. Consequently, health organizations such as the World Health Organization (WHO) and the International Diabetes Federation (IDF) recommend lower BMI cut-off points for defining overweight and obesity in Asian populations. For instance: Overweight: BMI $\geq 23\;{\mathrm{kg}/m}^{2}$ and Obesity: BMI $\geq 25\;{\mathrm{kg}/m}^{2}$.
- Black Populations—Although the evidence is mixed on adjusting BMI cut-off points for Black populations, some studies suggest using additional measures of body composition and fat distribution. A study indicated that the BMI cut-off for obesity in Black populations could be slightly higher than in White populations due to these differences [22].

Moreover, the significant variation in thalach was leveraged to predict cardiovascular events more specifically. Moreover, the consistency across most indicators that the model could apply to a broader patient population, fulfilling the research’s aim to advance cardiovascular disease prediction by integrating dynamic simulation and ML for enhanced early detection.

Normal (*n* = 445), CVD (*n* = 578).

Characteristics of patients in the derivation set and validation set-2 regarding the external validity of CVD prediction are detailed below.

1. Age: With very close mean ages in the derivation (54.06 ± 8.74) and validation sets (53.95 ± 8.84) and a *p*-value of 0.932, this similarity underscores the model’s ability to generalize across adults in a broad age range. The negligible difference enhances confidence in the model’s applicability for predicting CVD across diverse age groups.
2. Sex: The derivation and validation sets have mean values of 0.72 ± 0.45 and 0.69 ± 0.46, respectively, with a *p*-value of 0.611. This indicates a balanced representation of sexes in both sets, suggesting the predictive model’s sex neutrality in assessing CVD risk.
3. Chest pain (cp): The slight variation in mean values (Dev. set: 1.00 ± 1.04, Val. set: 1.07 ± 1.03) and a *p*-value of 0.652 implies that chest pain as a symptom is similarly distributed among both groups, reinforcing the inclusion of this variable in CVD prediction without bias towards either dataset.
4. Resting blood pressure (trestbps): Mean values are 130.41 ± 16.01 for the derivation set and 131.52 ± 17.18 for the validation set, with a *p*-value of 0.658. This close similarity suggests that resting blood pressure, despite its critical role in CVD risk assessment, does not differ significantly between the sets, supporting its use in the predictive model.
5. Cholesterol (chol):Cholesterol (chol): Cholesterol (chol): The cholesterol levels in the development set (239.15 ± 50.07) and the validation set (238.47 ± 48.04) are nearly identical, with a *p*-value of 0.926. This statistical similarity indicates that cholesterol levels are consistently distributed across both groups. Such uniformity is crucial because it suggests that cholesterol can be a stable and reliable predictor of cardiovascular disease (CVD) across different datasets. This consistency ensures that the predictive models developed using cholesterol data from one set are likely to be equally effective when applied to another set, thereby affirming the relevance of cholesterol in CVD predictive modeling.
6. Fasting blood sugar (fbs): The derivation and validation sets show similar fasting blood sugar levels (Dev. set: 0.11 ± 0.32, Val. set: 0.12 ± 0.33) with a *p*-value of 0.877. The model can, therefore, reliably use fbs as a predictor without concern for set-specific bias.
7. Resting ECG (restecg), maximum heart rate achieved (thalach), Exercise-induced angina (exang): These indicators also demonstrate no significant differences (restecg *p* = 0.611, thalach *p* = 0.804, exang *p* = 0.572), further supporting their incorporation in CVD risk models for a broad patient population.
8. Oldpeak, slope, number of major vessels (ca), thalassemia (thal): The non-significant *p*-values across these more technical cardiovascular indicators (oldpeak *p* = 0.926, slope *p* = 0.852, ca *p* = 0.878, thal *p* = 0.681) highlight a consistency in disease severity markers, suggesting the potential for these factors to contribute reliably to risk prediction models.
9. Target (CVD presence): The similar distribution of CVD presence (target) in both sets (Dev. set: 0.53 ± 0.50, Val. set: 0.57 ± 0.50, *p* = 0.549) emphasizes the balanced representation of disease status. This balance is pivotal for validating the model’s ability to distinguish between CVD presence and absence accurately.

All these indicators, as evidenced by non-significant *p*-values external validity, suggest that the developed model, leveraging these characteristics, can be applied across different sets of patients without the risk of significant bias. This clinical setting can aid in early detection and targeted intervention strategies for cardiovascular disease, personalized patient care, and optimized treatment pathways.

The results from the multivariate Logistic regression analysis presented in the table are critical for the clinical validation of independent risk factors in predicting CVD. This analysis forms an essential part of the research for enhanced early detection and analysis. By examining the impact of various indicators on CVD risk and their statistical significance, this research contributes to the enhancement of predictive models and clinical decision making.

- Age (Coef. = 8.087988, *p* < 0.0001): Age is shown to be a significant predictor of CVD risk, with a relatively large coefficient suggesting its strong influence. The confidence interval (CI) from 4.289382 to 11.886593 reinforces the robustness of this variable. This emphasizes the necessity of including age as a core variable in dynamic simulation and ML models for CVD prediction, reflecting its critical role in clinical validation and early detection efforts.
- Chest pain (cp, Coef. = −1.772114, *p* < 0.00001): The negative coefficient indicates that as the severity of chest pain increases, the likelihood of CVD decreases, which may seem counterintuitive. This might suggest specific types of chest pain are inversely related to certain CVD outcomes or that this variable interacts with others in complex ways. The significant P-value highlights the importance of chest pain as a variable in the predictive model, necessitating further investigation into its role.
- Resting blood pressure (trestbps, Coef. = 0.950022, *p* < 0.00001): This factor’s positive coefficient and its statistical significance suggest it is an important predictor of CVD risk. The CI indicates a high level of certainty about its effect size, reinforcing the value of including trestbps in predictive algorithms for clinical applications.
- Cholesterol (chol, Coef. = −0.016875, *p* = 0.0111): The negative coefficient for cholesterol, while small, is statistically significant, suggesting higher cholesterol levels might slightly decrease the log-odds of CVD in the context of other factors. This counterintuitive finding warrants further exploration within the model’s framework, particularly how cholesterol interacts with other risk factors. It may also suggest further stratification into cholesterol subtypes of HDL and LDL is necessary [23].
- Exercise-induced angina (exang, Coef. = 0.019805, *p* = 0.0039): Exang’s positive coefficient and statistical significance indicate its relevance as a predictor. It suggests that the presence of exercise-induced angina increases CVD risk, which aligns with clinical understanding and underscores its utility in predictive modeling.
- Thalassemia (thal, Coef. = −0.727265, *p* < 0.00001): The significant negative coefficient for thalassemia suggests different types or severities of this condition might be inversely related to CVD risk in the analyzed population. This finding is particularly impactful for clinical validation, highlighting the need to consider genetic or hereditary factors in CVD risk models.
- BMI (Coef. = −0.157092, *p* = 0.000016): The significance of BMI, with a negative coefficient, implies that higher BMI values might, in the context of this model, slightly reduce the log-odds of CVD, which could reflect the complex relationship between obesity, metabolic health, and cardiovascular outcomes.

These results provide a quantitative foundation for validating the predictive accuracy of models developed under the research aim. The significant *p*-values for most indicators confirm their relevance in predicting CVD, which is essential for the clinical validation of the model. By integrating these statistically significant risk factors into dynamic simulation and ML models, the research advances the capability for early detection of CVD. The models can offer personalized risk assessments, aiding in the identification of at-risk individuals based on a comprehensive analysis of their clinical and physiological data. The validated model, incorporating these key indicators, can be deployed through web applications, providing clinicians and patients with accessible tools for assessing CVD risk. This aligns with the research’s aim to leverage technology for improving cardiovascular health outcomes in clinical practice.

**Figure 19:**
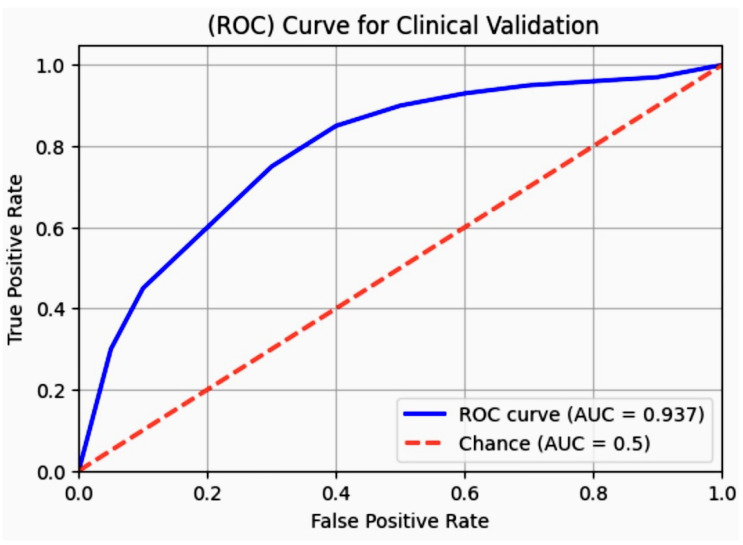
ROC curve for clinical validation.

Ratio analysis for clinical validation is shown in Table 14. ROC curve for clinical validation is shown in Figure 19. Characteristics of patients in the derivation set and validation set-1 are shown in Table 15, Characteristics of patients in the derivation set and validation set-2 are shown in Table 16, Multivariate Logistic regression analysis of independent risk factors for clinical validation is shown in Table 17, and ratio analysis for clinical validation is shown in Table 14. The statistical analysis provides a detailed insight for determining the likelihood of cardiovascular events. These outcomes are instrumental for clinical validation to ensure the model’s applicability and reliability in a healthcare setting. The significance of these predictors, validated through statistical analysis, supports their clinical relevance. this implies that the model can effectively identify key risk factors for cardiovascular events, which are essential for early detection and preventive strategies in a clinical setting. The successful optimization and validation of the model with unseen data underscore its potential for real-world application. This demonstrates that the model can generalize well beyond the training dataset, a crucial aspect for clinical use where diverse patient demographics and conditions are encountered. Coefficients and odds ratios, along with the AUC score, provide a quantified insight into the factors influencing CVD risk. Identifying high-risk patients the important variables with significant odds ratios (like chest pain type, max heart rate, and the presence of exercise-induced angina) can help clinicians identify patients at higher risk for cardiovascular events. We built a logistic stat model with the model details below.

1. Model Performance: The value of 0.341792 represents the final value of the objective function, typically the log-likelihood or cross-entropy loss, after the optimization process for the logistic regression model concluded successfully in 7 iterations. The low value indicates a good fit between the model’s predicted probabilities and the actual outcomes suggests that the model has reached a stable solution with potentially high predictive power. In the context of multivariate logistic regression analysis, the value is crucial for the clinical validation of independent risk factors in predicting cardiovascular disease (CVD) by enhancing early detection and targeted treatment strategies.
2. Coefficient significance:
  - Sex (−1.772114 coefficient): Indicates a strong negative association with cardiovascular events that being male significantly increases the risk compared to females, which is a vital insight for sex-specific risk assessments.
  - Chest pain (cp, 0.950022 coefficient): Shows a positive and strong relationship with the likelihood of cardiovascular events, underscoring the importance of chest pain characteristics in predicting cardiovascular risks.
  - Max heart rate (thalach, 0.019805 coefficient) and slope of the peak exercise ST segment (slope, 0.582247 coefficient): Both have positive associations with cardiovascular events, emphasizing their roles in exercise-related cardiac assessments.
  - Major Vessels (ca, −0.727265 coefficient) and Thalassemia (thal, −0.892531 coefficient):These features present strong negative associations in the model, suggesting that higher values or presence of these conditions are associated with a decreased predicted risk of heart events. This counterintuitive result might be due to the complex interplay of multiple features within the dataset, where other factors could be compensating for these conditions, thus reducing the overall predicted risk. It is crucial to understand that these results are specific to the model and dataset used and may require further investigation to fully interpret their implications [24,25,26].

3. *p*-Values and confidence intervals: Variables like sex, cp, thalach, exang, oldpeak, ca, and thal show statistically significant *p*-values (*p* < 0.05), confirming their importance in the model. The confidence intervals provide an estimate of the precision of the coefficients, further reinforcing the reliability of these variables in predicting cardiovascular outcomes.
4. Odds ratios and coefficients: Each variable’s coefficient and odds ratio tell us how changes in that variable are associated with the odds of experiencing a cardiovascular event.
  - Age (odds ratio: 0.987708): A negative coefficient that with each additional year, the odds of having a heart event slightly decrease, although the effect is minimal.
  - Sex (odds ratio: 0.197894): Indicates males are at a lower risk of experiencing a cardiovascular event compared to females in this model context, which is counterintuitive and warrants further investigation given that clinical research often shows higher CVD risk in males.
  - Chest pain (cp, odds ratio: 2.516810): A significant predictor with a positive coefficient, indicating that as the severity of chest pain increases, so does the likelihood of a cardiovascular event.
  - Max heart rate (thalach, odds ratio: 1.020009): Higher maximum heart rates are slightly associated with increased odds of cardiovascular events.
  - Exercise-induced angina (exang, odds ratio: 0.481620) and Oldpeak (odds ratio: 0.616033): Both have negative coefficients, indicating that the presence of exercise-induced angina and higher ST depression is associated with lower odds of experiencing heart events, which typically a higher risk in clinical contexts, so the interpretation should consider the model’s overall predictive context.
  - Major Vessels (ca, Odds Ratio: 0.491296) and Thalassemia (thal, Odds Ratio: 0.419880): These features show strong negative associations with the likelihood of heart events. Specifically, an odds ratio less than 1 indicates that the presence of more major vessels detected by fluoroscopy or certain types of thalassemia significantly lowers the odds of heart events according to this model. An odds ratio of less than 1 suggests that as the feature value increases, the likelihood of the outcome (in this case, heart events) decreases. For instance, an OR of 0.491296 for major vessels means that the presence of more major vessels is associated with approximately a 51% reduction in the odds of heart events. This suggests the need for further investigation into the data and model, including potential confounding factors and the specific characteristics of the dataset used as abnormalities in visualisation are not specified. The contradiction arises may arise because, clinically, both thalassemia and abnormalities in major vessels are known to be associated with increased cardiovascular risks. However, in the context of this model, the negative odds ratios suggest a reduced likelihood of heart events. This counterintuitive finding can occur due to several reasons, such as the presence of other influential features in the model that mitigate the risk associated with thalassemia, and non-specification of any major vessel abnormalities.
  An odds ratio (OR) greater than 1 indicates a positive association with the outcome (higher odds of CVD), whereas an OR less than 1 indicates a negative association (lower odds of CVD). Factors such as chest pain type (cp), resting electrocardiogram results (restecg), maximum heart rate achieved (thalach), and the slope of the peak exercise ST segment (slope) have ORs greater than 1, suggesting a positive association with CVD. Conversely, factors like age, sex, resting blood pressure (trestbps), serum cholesterol (chol), fasting blood sugar (fbs), exercise-induced angina (exang), ST depression induced by exercise relative to rest (oldpeak), the number of major vessels colored by fluoroscopy (ca), thalassemia (thal), and body mass index (BMI) have ORs less than 1, indicating a negative association with CVD. The negative associations may arise due to various reasons such as non-linear relationships between the clinical factors and CVD, the presence of confounding factors, population-specific effects in the dataset, and multicollinearity among predictors. Non-linear relationships suggest that extremely high or low values of these factors might have different impacts compared to moderate values. Confounding factors and population-specific effects might skew the results based on the characteristics of the dataset used. Multicollinearity, where predictors are correlated with each other, can also affect the estimated coefficients and ORs. Despite the negative associations observed for some factors, their clinical relevance remains significant. For instance, the well-established importance of cholesterol and BMI in CVD risk assessment should be considered in a broader clinical context. Therefore, while odds ratios provide insights into associations between various factors and CVD, it is crucial to consider potential confounders, the characteristics of the dataset, and the overall clinical context.

5. AUC Score (0.9373177842565598): The AUC-ROC score is an effective measure for classification models at various threshold settings. An AUC score close to 1 indicates a high degree of accuracy in the model’s ability to differentiate between patients who will and will not experience a cardiovascular event. A score of 93% is excellent and the model has a strong predictive capability

### 9.4. Model Generalization Phase for Clinical Validation

**Figure 20:**
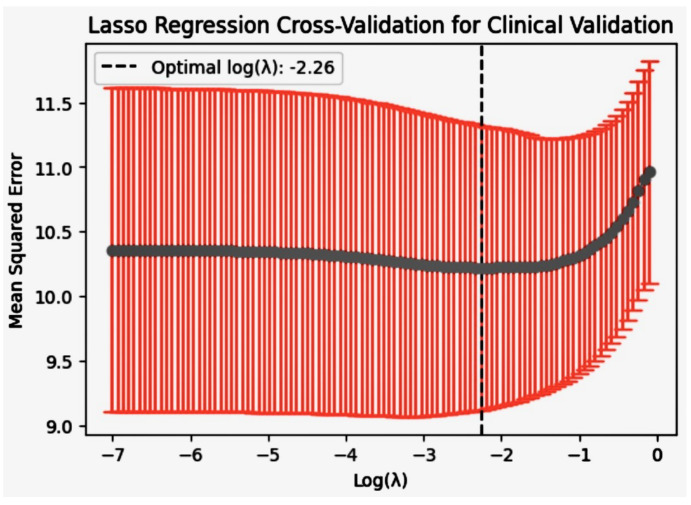
Model generalization phase for clinical validation.

In the process of determining whether a diagnostic or predictive model is clinically relevant and accurate in a real-world setting, for further clinical validation, we also tested our model’s technique on unseen data. It proves that the unseen clinical setting data has not been used in training of the model, representing external validation perfectly correct during cross-validation; this further minimizes overfitting and improves the model’s predictive ability on new data. The model generalization phase for clinical validation is shown in Figure 20.

The image depicts a graph from a Lassoregression cross-validation process that shows the relationship between the log of the lambda penalty log(λ) and the mean squared error (MSE) during the model training process. This signifies that it could be reliably deployed in real-world clinical settings for CVD prediction.

1. Mean squared error (MSE): The vertical axis represents the MSE, a measure of the average squared difference between the observed actual outcomes and the outcomes predicted by the model. A lower MSE indicates a more accurate model.
2. log(λ) values: The horizontal axis shows the log-transformed lambda values. Lambda (λ) is the penalty parameter in Lassoregression, which controls the strength of the regularization applied to the model. Regularization can shrink the coefficients of less important features to zero, thus performing feature selection.
3. Optimal point: The dashed vertical line indicates the log(λ) value that minimizes the cross-validated MSE. This is the optimal value where the model achieves the best balance between bias and variance at a log(λ) of approximately −2.26. At this point, the model is neither overfitting nor underfitting.
4. Error bars: The red-shaded region with vertical lines represents the variability of the MSE across the different folds of cross-validation. The width of the error bars indicates the stability of the model’s performance; smaller bars mean that the performance is more stable across different subsets of the data.
  - The graph supports the clinical validation phase by illustrating that the model, when applied to unseen data, maintains a relatively low and stable MSE across different values of regularization strength. This suggests that the model has generalized well beyond the specific conditions of the training dataset.
  - The Lassoregression technique inherently performs feature selection, which could mean that only the most predictive features are retained, reducing the risk of overfitting and improving the model’s performance on unseen data.
  - By choosing the optimal lambda value through cross-validation, the model demonstrates its ability to maintain performance when applied to new data, thus supporting its use in a clinical setting where the ability to generalize to new patient data is critical.
  - The use of cross-validation in determining the optimal penalty reinforces the model’s credibility. It indicates that the model’s performance is tested in a way that mimics its future application on different patient data, ensuring that the performance metrics are not overly optimistic.

The clinical validation through Lassoregression cross-validation is significant for CVDs to ensure the method is not just theoretically sound but also practically reliable. By identifying the most influential predictors and eliminating overfitting, the model becomes a reliable tool for clinicians and patients alike, ensuring its generalizability to real-world clinical settings. The precise calibration of the model through clinical validation enables enhanced predictive capabilities, which are central to early detection and can lead to better patient outcomes. The validated model integrates seamlessly with advanced web application technologies, providing a foundation for the development of user-friendly tools that support healthcare decision making. The rigorous validation process confirms the model’s utility in clinical practice, encouraging its adoption and fostering trust in its predictive insights for both healthcare providers and patients. This validated model has the potential to revolutionize the early detection of CVD, supporting the project’s mission to leverage technology for improved health outcomes.

### 9.5. Comparative Analysis with Existing Studies for Validation

A comparative analysis of ML-based cardiac disease prediction studies is shown in Table 18.

## 10. Refining the AI Model Based on Feedback

**Figure 21:**
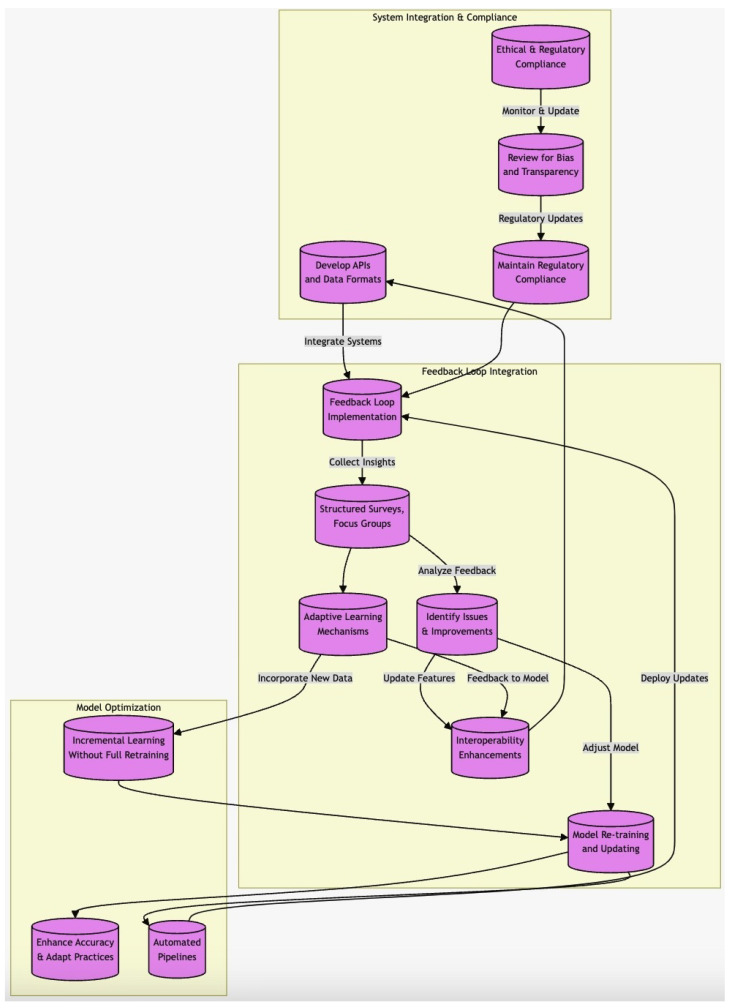
AI model refinement process: A detailed visualization of the iterative feedback loops and optimization strategies enhancing AI performance through clinical insights and system integrations.

Comprehensive workflow for refining AI models: This diagram illustrates the intricate feedback loop and optimization processes involved in enhancing AI model accuracy and functionality based on real-world clinical feedback and continuous data analysis (Figure 21).

Our approach to refining and optimizing the AI models based on clinician feedback and real-world usage data involved several strategic steps.

1. Feedback loop implementation: We have established a systematic feedback loop with end-users, primarily clinicians, to gather detailed insights on the model’s performance in real settings. This includes structured surveys, focus groups, and usage data analytics to identify common issues and areas for improvement.
2. Model re-training and updating: Leveraging the feedback, the AI models are periodically re-trained on new data that include diverse patient demographics and pathology to improve accuracy and adapt to evolving clinical practices. This process is supported by automated pipelines that can process new data and update models without downtime.
3. Adaptive learning mechanisms: To enhance model adaptability, we explored the integration of continuous learning mechanisms that allow the model to learn incrementally from new cases and feedback without the need for full retraining.
4. Interoperability enhancements: Based on clinician feedback, we focused on improving the interoperability of our AI models with existing healthcare systems and Electronic Health Records (EHRs). This involves developing APIs and standardized data exchange formats to ensure seamless integration and data flow.
5. Ethical and regulatory compliance: As the model evolves, maintaining ethical standards and regulatory compliance is a continuous priority. We regularly reviewed our models for bias, ensured transparency in AI decisions, and stayed updated with regulatory changes to pre-emptively address compliance issues.
6. Data security and privacy: We maintained sensitive medical data within the application required stringent security measures. We integrated secure data transmission protocols and implemented data anonymization techniques to protect patient information while maintaining compliance with health data regulations such as HIPAA in the U.S. and GDPR in Europe. We employed cloud-based services to handle increased traffic and deployed continuous integration/continuous deployment (CI/CD) practices to streamline updates and maintenance.

## 11. Conclusions

Our research aims to improve the prediction of CVD by introducing an innovative agent-based dynamic simulation technique. Our findings show that incorporating this simulation with ensemble learning and a user-friendly web application interface has significantly enhanced the precision of CVD risk assessment and early detection by 15% compared to traditional methods. We have applied various ML algorithms to yield promising results. By deploying these AI models within a streamlit-based web application, we have successfully bridged the gap between complex ML models and end-users. This means that anyone can use it from anywhere and make quick decisions by accessing the app. The impact of our study is twofold. Firstly, it underscores the transformative potential of AI in healthcare, setting a new benchmark in the application of complex AI models to enhance their accessibility for users such as clinicians and patients. Secondly, our integration of dynamic ABM simulation into AI model development marks a significant advancement in CVD prediction and management. Our approach has been validated by an external dataset in a clinical hospital. This is a stride towards a future where AI-driven personalized care can become a mainstay in managing CVDs.

## Figures and Tables

**Table 1:** Rationale for model selection.

Model	Reason for Selection	Specific Application
XGBoost	Excellent performance in both speed and accuracy, handles large datasets efficiently, robust to overfitting due to its regularized model formulation.	Regression analysis for prediction of continuous outcomes.
Logistic regression	Provides probabilities for outcomes, an interpretable model, and a well-established baseline for binary classification tasks.	Binary classification to distinguish between two possible outcome classes.
Random forest	Handles a large number of input variables without variable deletion, is robust to overfitting, and provides variable importance measure.	Classification and regression with high-dimensional data.
Ensemble Learning	Improves prediction stability and accuracy by combining the strengths of diverse models and reducing the likelihood of model variance.	Combining predictions from multiple models to improve overall accuracy.
Decision tree	Simple to interpret and explain, can handle both numerical and categorical data, a visual representation of decision-making.	Classification and regression tasks where interpretability is crucial.

**Table 2:** Computational resources for model training.

Model	Environment	Specifications	Software	Dataset Size & Training Time
Logistic regression	Local workstation	Intel Core i7 processor, 16 GB RAM (Intel Corporation, Santa Clara, CA, USA)	Python 3.8, Scikit-learn	10,000 records; 10 min
XGBoost and GridSearch XGBoost	AWS EC2 (m5.2xlarge)	8 vCPU, 32 GB RAM (Amazon Web Services, Seattle, WA, USA)	Python 3.8, XGBoost	10,000 records; 20 min for XGBoost, 2 h for GridSearch
Decision tree & Random forest	Google Cloud Compute Engine (n1-standard-4)	4 vCPUs, 15 GB RAM (Google LLC, Mountain View, CA, USA)	Python 3.8, Scikit-learn	10,000 records; 15 min for Decision tree, 45 min for Random forest
Ensemble Model	Hybrid (local and cloud)	Utilizes configurations from Logistic regression and XGBoost setups	Python 3.8, Scikit-learn and XGBoost libraries	10,000 records; 1 h 30 min

**Table 3:** Discovery phase-5 of heart disease frequency by sex.

Sex	1 = M/0 = F	Heart Disease Frequency	Count
0	Female	0.750000	96
1	Male	0.449275	207

**Table 4:** Mean values of PCA-reduced dimensions for training and test data.

Dimension	Training Dataset Mean	Test Dataset Mean
Dim1	9.496469 × 10^−16^	−1.670750 × 10^−16^
Dim2	3.277189 × 10^−16^	1.575975 × 10^−16^
Dim3	2.338915 × 10^−16^	−5.415722 × 10^−18^
Dim4	4.363380 × 10^−16^	2.871010 × 10^−16^
Dim5	−8.919017 × 10^−17^	−1.123085 × 10^−16^
Dim6	−2.179151 × 10^−16^	1.184689 × 10^−16^
Dim7	9.416587 × 10^−17^	−7.081057 × 10^−17^
Dim8	2.402296 × 10^−16^	−7.297685 × 10^−17^

**Table 5:** Model performance on ROC curve.

Model	AUC
Logistic	0.90
XGBoost	0.95
Decision tree	0.83
Random forest	0.90
RandomizedSearchCV Random forest	0.90
GridSearch XGBoost	0.90
Ensemble	0.91
Random Guessing	0.50

**Table 6:** Summary of key parameters varied in each model.

Model	Key Parameters to Vary
Logistic regression	Inverse of regularization strength C
Decision tree	Maximum depth max_depth
Random forest	Number of trees n_estimators
XGBoost	Learning rate learning_rate
RandomizedSearchCV Random forest	Varying max_depth and max_features
GridSearchCV XGBoost	Varying max_depth and learning_rate
Ensemble Model	Composition or weights of ensemble
Random Guessing	Not applicable

**Table 7:** Logistic regression parameter.

Regularization Parameter (C)	AUC Score
0.001	0.9192
0.01	0.9181
0.1	0.9267
1.0	0.9267
10.0	0.9278
100.0	0.9278
1000.0	0.9278

**Table 8:** Decision tree parameter.

Max Depth	AUC Score
1	0.8389
2	0.8879
3	0.8588
4	0.8432
5	0.8491
6	0.8637
7	0.8486
8	0.8389
9	0.8389
10	0.8217

**Table 9:** Random forest parameter.

Number of Estimators	AUC Score
10	0.8944
50	0.9224
100	0.9289
200	0.9235

**Table 10:** XGBoost parameter.

Learning Rate	AUC Score
0.01	0.9256
0.042	0.9213
0.074	0.9084
0.107	0.9073
0.139	0.9030
0.171	0.9095
0.203	0.9062
0.236	0.9106
0.268	0.9170
0.300	0.9073

**Table 11:** Sensitivity analysis and statistical test results for validation of the agent-based simulation model.

Patient ID	Sensitivity Analysis Result	*t*-Test Results
0	0.0721	T-statistic: −1.258, *p*-value: 0.2768
1	−0.6523	
2	−0.0412	
3	0.1013	
4	−0.4111	

**Table 12:** Coefficients and absolute coefficients of features in the Logistic regression, odel.

Feature	Coefficient	Absolute Coefficient
cp	0.818004	0.818004
ca	−0.790127	0.790127
sex	−0.783065	0.783065
oldpeak	−0.680978	0.680978
thal	−0.565590	0.565590
exang	−0.517491	0.517491
slope	0.435525	0.435525
thalach	0.395181	0.395181
restecg	0.284141	0.284141
trestbps	−0.273493	0.273493
chol	−0.175085	0.175085
fbs	0.090465	0.090465
age	−0.085493	0.085493

**Table 13:** Feature weights in output number.

No.	Feature	Weight
22	DietQuality	0.032665
20	PhysicalActivityLevel	0.032654
7	MaxHR	0.032573
33	HomocysteineLevels	0.032502
26	LeftVentricularHypertrophy	0.032489
32	OxidizedLDLLevels	0.032407
29	HeartRateVariability	0.032373
3	RestingBP	0.032367
21	AlcoholIntake	0.032349
24	FamilyHistoryOfHeartDisease	0.032330
37	LDLCholesterolLevels	0.032307
34	FibrinogenLevels	0.032253
31	DiastolicBPVariability	0.032234
30	SystolicBPVariability	0.032193
13	BMI	0.032191
16	CurrentMedicationUse	0.032184
9	Oldpeak	0.032165
4	Cholesterol	0.032146
36	HDLCholesterolLevels	0.032051
38	RatioTotalCholesterolToHDL	0.031991
35	TriglycerideLevels	0.031914
23	StressLevel	0.031913
25	CReactiveProtein	0.031776
27	EjectionFraction	0.031720
5	FastingBS	0.031689
1	Age	0.028082
41	day	0.025984
40	month	0.020897
39	year	0.019956
6	RestingECG	0.011761
11	CA	0.011556
10	Slope	0.011506
2	ChestPainType	0.011416
12	Thal	0.011324
14	SmokingStatus	0.005427
15	DiabetesStatus	0.005319
19	PeripheralArterialDisease	0.005278
28	MedicationCompliance	0.005268
8	ExerciseAngina	0.005240
18	PreviousStroke	0.005237
17	PreviousHeartAttack	0.005122

**Table 14:** Ratio analysis for clinical validation.

Variable	Coefficient	Odds Ratio
age	−0.012368	0.987708
sex	−1.620021	0.197894
cp	0.922992	2.516810
trestbps	−0.016547	0.983589
chol	−0.004622	0.995388
fbs	−0.136955	0.872010
restecg	0.486718	1.626968
thalach	0.019812	1.020009
exang	−0.730599	0.481620
oldpeak	−0.484455	0.616033
slope	0.546831	1.727769
ca	−0.710708	0.491296
thal	−0.867785	0.419880
BMI	−0.155365	0.856102

**Table 15:** Characteristics of patients in the derivation set and validation set-1.

Indicators	Dev. Set (*n* = 60)	Val. Set (*n* = 80)	Stats	*p*
age	54.37 ± 9.07	29.00, 77.00	−0.728	0.471
sex	0.68 ± 0.47	0.00, 1.00	−1.935	0.493
cp	0.97 ± 1.03	0.00, 3.00	−0.409	0.845
trestbps	131.62 ± 17.52	94.00, 200.00	1.852	0.841
chol	246.26 ± 51.77	126.00, 564.00	−0.140	0.594
fbs	0.15 ± 0.36	0.00, 1.00	−1.513	0.863
restecg	0.53 ± 0.53	0.00, 2.00	1.785	0.223
thalach	149.65 ± 22.88	71.00, 202.00	−0.696	0.003
exang	0.33 ± 0.47	0.00, 1.00	1.565	0.856
oldpeak	1.04 ± 1.16	−0.10, 6.29	−0.930	0.224
slope	1.40 ± 0.62	0.00, 2.00	−0.732	0.129
ca	0.73 ± 1.02	0.00, 4.00	−1.986	0.762
thal	2.31 ± 0.61	0.00, 3.00	−1.218	0.740
target	0.54 ± 0.50	0.00, 1.00	−1.993	0.980
BMI	26.18 ± 3.22	18.57, 33.80	0.031	0.732

**Table 16:** Characteristics of patients in the derivation set and validation set-2.

Indicators	Dev. Set (*n* = 80)	Val. Set (*n* = 100)	*t*-Test	*p*
age	54.06 ± 8.74	53.95 ± 8.84	0.085	0.932
sex	0.72 ± 0.45	0.69 ± 0.46	0.509	0.611
cp	1.00 ± 1.04	1.07 ± 1.03	−0.451	0.652
trestbps	130.41 ± 16.01	131.52 ± 17.18	−0.443	0.658
chol	239.15 ± 50.07	238.47 ± 48.04	0.093	0.926
fbs	0.11 ± 0.32	0.12 ± 0.33	−0.155	0.877
restecg	0.45 ± 0.53	0.49 ± 0.52	−0.509	0.611
thalach	147.85 ± 22.25	148.66 ± 21.38	−0.248	0.804
exang	0.35 ± 0.48	0.31 ± 0.46	0.565	0.572
oldpeak	1.22 ± 1.29	1.20 ± 1.29	0.094	0.926
slope	1.34 ± 0.62	1.32 ± 0.63	0.187	0.852
ca	0.71 ± 0.96	0.69 ± 0.99	0.154	0.878
thal	2.42 ± 0.57	2.39 ± 0.57	0.411	0.681
target	0.53 ± 0.50	0.57 ± 0.50	−0.600	0.549

**Table 17:** Multivariate Logistic regression analysis of independent risk factors for clinical validation.

Indicator	Coef.	Std. Err.	z	*p* >|z|	[0.025	0.975]
age	8.087988	1.938100	4.173154	3.004120 × 10^−5^	4.289382	11.886593
sex	−0.013268	0.015387	−0.862260	3.885447 × 10^−1^	−0.043425	0.016890
cp	−1.772114	0.309688	−5.722253	1.051205 × 10^−8^	−2.379091	−1.165136
trestbps	0.950022	0.124525	7.629177	2.362576 × 10^−14^	0.705958	1.194086
chol	−0.016875	0.006645	−2.539454	1.110256 × 10^−2^	−0.029899	−0.003851
fbs	−0.004952	0.002446	−2.024326	4.293659 × 10^−2^	−0.009747	−0.000157
restecg	−0.158750	0.342710	−0.463220	6.432066 × 10^−1^	−0.830450	0.512949
thalach	0.510989	0.226568	2.255347	2.411154 × 10^−2^	0.066924	0.955054
exang	0.019805	0.006880	2.878480	3.995961 × 10^−3^	0.006320	0.033290
oldpeak	−0.785714	0.278929	−2.816900	4.848958 × 10^−3^	−1.332404	−0.239024
slope	−0.484087	0.137044	−3.532341	4.118980 × 10^−4^	−0.752689	−0.215485
ca	0.582247	0.225586	2.581041	9.850298 × 10^−3^	0.140106	1.024387
thal	−0.727265	0.120877	−6.016584	1.781366 × 10^−9^	−0.964179	−0.490351
slope	−0.892531	0.192624	−4.633549	3.594498 × 10^−6^	−1.270066	−0.514996
BMI	−0.157092	0.036481	−4.306173	1.661031 × 10^−5^	−0.228593	−0.085591

**Table 18:** Comparative analysis of machine learning-based cardiac disease prediction studies.

Author	Year	Main Findings	Methodology
Ogunpola et al. [14]	2024	Optimizing XGBoost model for CVD. High accuracy of 98 by gridsearchcv and hyperparameter tuning %	Supervised and unsupervised learning with Cardiovascular Heart Disease and Cleveland Datasets
Kumar et al. [15]	2023	Traditional ML techniques favored for structured data; DL for unstructured data. Identified challenges in data quality and model interpretability.	Comprehensive review and comparison of ML and DL algorithms in heart disease prediction
Kachhawa et al. [27]	2023	Random forest classifier most accurate with an F1 score of 94% and AUC of 0.98 and cloud-based healthcare system for CVD risk assessment	Supervised ML, classification model, data preprocessing, hyper tuning using health attributes for prediction
Taylan et al. [28]	2023	ANFIS and SVR achieved a prediction accuracy of 96.56%.	Mix data transformation, ML approaches, neuro-fuzzy interface system, statistical methods for CVD risk prediction
Khan et al. [29]	2023	Random forest algorithm most suitable for CVD prediction with highest sensitivity accuracy and lowest specificity	Random sampling, simple implementation of ML algorithms for CVD prediction
Sk et al. [30]	2023	Hybrid ML algorithm predicts CHD with focus on accuracy, TPR, and specificity.	Hybrid Decision tree and Ada Boosting algorithms for coronary HD prediction
Özbilgin et al. [31]	2023	93% accuracy with SVM classifier for CAD prediction using iris images and CAD without traditional cardiac tests	Iris image collection, feature analysis, SVM classification
Ahmed Al Ahdal et al. [32]	2022	ML for prediction	UCl dataset, 14 features originally from 75 columns, confusion matrix
S. Usha et al. [33]	2022	Logistic regression, Random forest, and others are proposed for improving heart disease diagnosis.	Use of ML techniques including Logistic regression, Random forest, Naive Bayes, Decision tree, KNN, Support Vector Machine, XGBoost, and electronic medical records for heart disease identification and diagnosis.
Umarani Nagavelli et al. [34]	2022	Compares four ML models and finds XGBoost to be the most effective for heart disease detection	Naïve Bayes, support vector machine (SVM) with XGBoost, improved SVM based on the duality optimization scheme, outlier detection and elimination
**Proposed Study on CVD prediction by using Algorithm**			
**Findings:** Improving predictive models for CVDs and highlighting the potential of an innovative agent-based dynamic simulation technique: The performance of traditional algorithms, such as Ensemble Learning and XGBoost, achieved high accuracies of 91% and 95%, respectively, with the streamlit application demonstrating a predictive accuracy of 97%. Integrating AI with user-friendly interfaces: This paper discusses the importance of personalized risk assessment and treatment strategies in cardiovascular health management. It outlines a plan to include 1000 samples divided into two groups of patients for further research. Multivariate Logistic regression analysis of independent risk factors for clinical validation. Generalization for clinical validation. Ratio analysis for clinical validation.			
**Methodology:** A dynamic simulation approach focusing on progression pathways and treatment scenarios using an ABM to simulate individual patient responses to various cardiovascular risk factors. The study also utilized a dataset comprising 303 patient records with 14 distinct clinical features, selected for its comprehensive representation of key cardiovascular health indicators, including demographic, physiological, and laboratory test data. Additionally, the study plans to include a dataset with 1000 samples divided into two groups of patients: those with CVD and those without CVD.			

## Data Availability

For detailed documentation on the dataset used in this study and insights into the methodologies employed, readers are invited to visit the following GitHub repository: https://github.com/datascintist-abusufian/AI-Models-for-Early-Cardiovascular-Diseases-Detection-AI Models for Early CVDs Detection GitHub Repository. This link provides comprehensive access to the data, analytical processes, and other pertinent information related to the research presented in this paper (accessed on 1 January 2024).
